# Comprehensive Analysis of Bacteriocins Produced by the Hypermucoviscous Klebsiella pneumoniae Species Complex

**DOI:** 10.1128/spectrum.00863-23

**Published:** 2023-05-08

**Authors:** Mi Nguyen-Tra Le, Thao Huu-Huong Nguyen, Van Minh Trinh, Tam Phuc-Bao Nguyen, Miki Kawada-Matsuo, Shizuo Kayama, Motoyuki Sugai, Hitoshi Komatsuzawa

**Affiliations:** a Department of Bacteriology, Hiroshima University Graduate School of Biomedical and Health Sciences, Hiroshima, Japan; b Project Research Center for Nosocomial Infectious Diseases, Hiroshima University, Hiroshima, Japan; c Antimicrobial Resistance Research Center, National Institute of Infectious Diseases, Tokyo, Japan; Institut de Recherche pour le Développement

**Keywords:** *Klebsiella pneumoniae*, *Klebsiella pneumoniae* species complex, antimicrobial peptide, bacteriocin, microcin, colicin

## Abstract

Klebsiella pneumoniae produces several kinds of bacteriocins that have antimicrobial effects against closely related species, but few studies have comprehensively reported bacteriocin distribution among the Klebsiella population. In this study, we identified bacteriocin genes in 180 K. pneumoniae species complex genomes, including 170 hypermucoviscous isolates, and investigated the antibacterial activity against 50 strains, including antimicrobial-resistant organisms, belonging to multiple species, namely, Klebsiella spp., Escherichia coli, Pseudomonas spp., Acinetobacter spp., Enterobacter cloacae, Stenotrophomonas maltophilia, Chryseobacterium indologenes, Staphylococcus aureus, Staphylococcus epidermidis, and Streptococcus mutans. Our study determined that 32.8% (59/180) of isolates carried at least one bacteriocin type. Different types of bacteriocin were usually present in different specific sequence types (STs); meanwhile, bacteriocins were not detected in certain STs. Microcin E492 was the most prevalent bacteriocin (14.4%), mostly in ST23 isolates, and displayed a wide spectrum of activity, including against Klebsiella spp., E. coli, Pseudomonas spp., and Acinetobacter spp. Cloacin-like bacteriocin was detected in 7.2% of strains, all of which were non-ST23 isolates, and exhibited inhibitory activity against closely related species, mainly Klebsiella spp. Klebicin B-like bacteriocin was detected at a rate of 9.4%, although 82.4% of these strains carried a disrupted bacteriocin gene, and an inhibitory effect could not be observed from the intact-gene-carrying isolates. Other bacteriocins, such as microcin S-like, microcin B17, and klebicin C-like, were detected at lower rates and had limited inhibitory activity. Our findings suggested that Klebsiella strains that carry different bacteriocin types may affect the composition of the surrounding bacterial community.

**IMPORTANCE** Klebsiella pneumoniae is a Gram-negative commensal bacterium that asymptomatically colonizes human mucosal membranes, such as the intestinal tract, but it is also a leading cause of health care- and community-associated infections. Additionally, multidrug-resistant K. pneumoniae has been continuously evolving, which significantly challenges the available chemotherapeutic treatment for its infections. K. pneumoniae produces several kinds of antimicrobial peptides known as bacteriocins, which have antibacterial activity against closely related species. This work was the first comprehensive report of bacteriocin distribution among the hypermucoviscous K. pneumoniae species complex population and the inhibitory activity of each bacteriocin type against various species, including multidrug-resistant strains. Our findings provide a foundation for future studies on the K. pneumoniae species complex, including studies on the competition within the microflora and the potential applications of bacteriocins in treating multidrug-resistant bacteria.

## INTRODUCTION

Klebsiella pneumoniae species complex (KpSC), which belongs to the *Enterobacteriaceae* family, is a natural inhabitant of the gastrointestinal microflora of humans but is also involved in extraintestinal infections, including urinary tract infections, cystitis, pneumonia, and surgical wound infections (1). KpSC is also an important cause of life-threatening infections, such as endocarditis and septicemia, and serious community-acquired infections, such as pyogenic liver abscesses and endogenous endophthalmitis (2). Furthermore, the risk of global dissemination of multidrug-resistant (MDR) and hypervirulent KpSC has become a recognized global threat (3, 4).

Bacteria produce a wide variety of toxins to compete and colonize local environments, especially when space and nutrition are limited. Bacteriocins are narrow-spectrum peptides that have been described as “the microbial weapon of choice” and have been used as a model for evolutionary and ecological investigations (5). Bacteriocins produced by Gram-negative bacteria are classified into the following two main families: low-molecular-mass peptides (below 10 kDa) termed microcins and high-molecular-weight proteins (30 to 80 kDa) termed colicins (6, 7).

Microcins are non-SOS-inducible, low-molecular-weight (less than 10 kDa), ribosomally synthesized peptides involved in competitive interactions between *Enterobacteriaceae* in the gut flora (8). They were reported to regulate microbial communities by influencing the bacterial interactions inside microbial ecosystems (9). They are divided into two categories according to molecular mass, disulfide bonds, and posttranslational modifications (10). Class I microcins are low-molecular-weight (<5 kDa) posttranslationally modified peptides, such as microcins B17, C7/C51, and J25, produced by Escherichia coli, while class II microcins are larger peptides (5 to 10 kDa). Class II is further divided into two subclasses, class IIa, some of which have disulfide bonds but no further posttranslational modification, and class IIb, which are linear peptides with posttranslational modifications at the C terminus (6). Examples of class IIa peptides include microcins MccL, MccV, and Mcc24, produced by E. coli, and examples of class IIb include microcin MccE492, produced by K. pneumoniae, and microcins MccM and MccH47, produced by E. coli. Their mechanisms of action include pore formation, nuclease activity (DNase and RNase), and inhibition of protein synthesis or DNA replication (6).

Colicins are bacteriocins produced by some E. coli strains that have antibacterial activity against closely related species (7). Gram-negative bacteria have been shown to produce several colicin-like bacteriocins, which were named after the species or genus, e.g., pyocins of Pseudomonas aeruginosa, cloacins of Enterobacter cloacae, pesticins of Yersinia pestis, marcescins from Serratia marcescens, or klebocins or klebicins of Klebsiella species. These are SOS-inducible high-molecular-weight bacteriocins with varied mechanisms of action, including pore formation (colicins E1, 5, 10, K, Ia, Ib, A, B, N, 683, and 647), DNase activity (klebicin B), rRNase activity (colicins E3, E4, and E6, cloacin DF13, and klebicins CCL and C), tRNase activity (colicins E5 and D and klebicin D), or cell wall disruption (11). Colicin-encoding genes are found on plasmids with a high frequency in natural populations of E. coli, suggesting their significance in microbial ecology.

Although studies have focused on bacteriocins produced by the E. coli population, there is a lack of comprehensive analysis on bacteriocins among Klebsiella spp. In this study, we determined the distribution of bacteriocins among 180 KpSC isolates, including 170 hypermucoviscous (HMV) strains and investigated the antimicrobial activity of bacteriocin-positive strains against various bacterial species, including MDR bacteria.

## RESULTS

### Characterization of the studied KpSC population.

**FIG 1:**
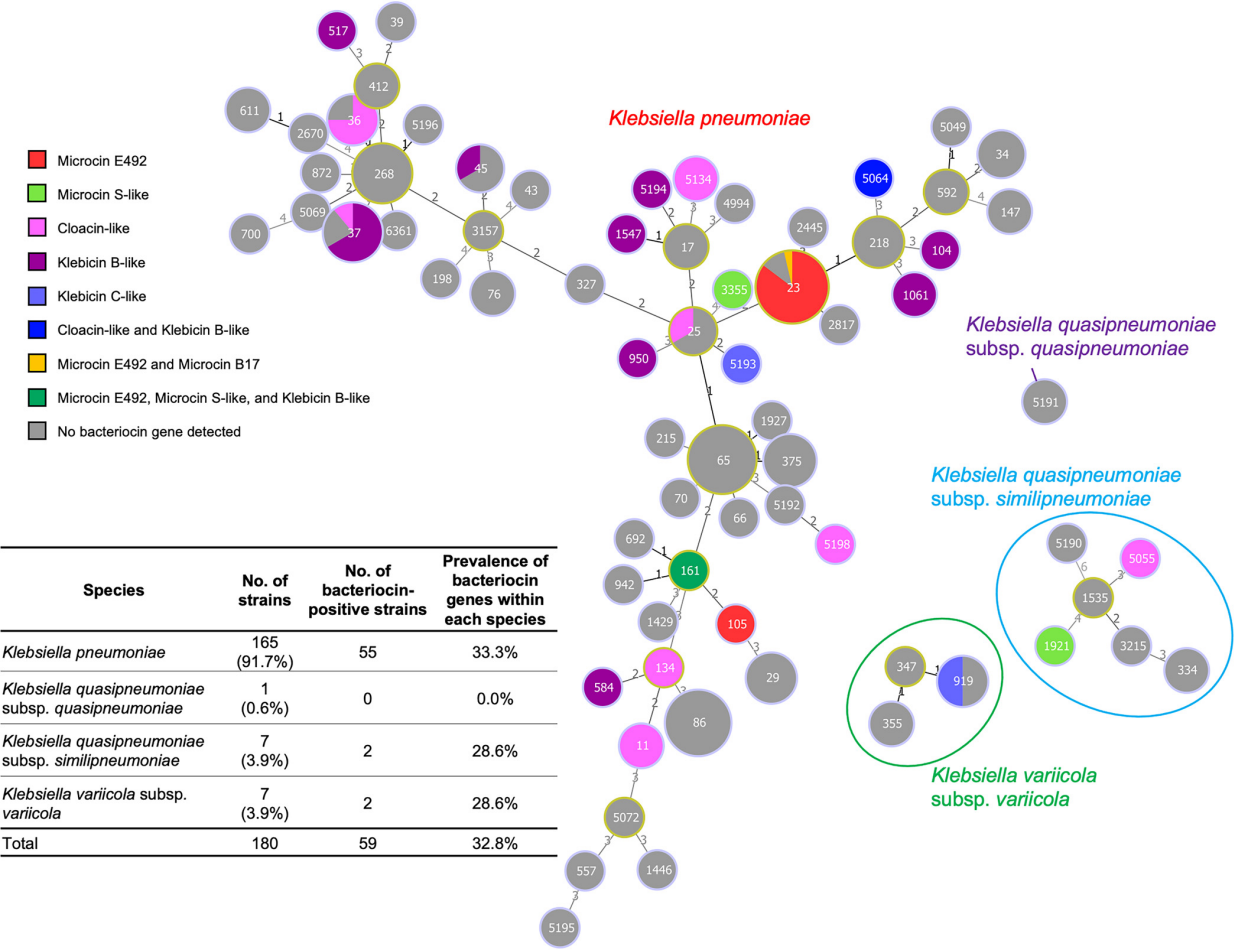
MST of 72 STs identified from our collection of 180 isolates. Each circle represents an ST, and the ST number is indicated in the middle of each circle. The size of the circle correlates with the number of isolates of that ST. Yellow-circle outlines indicate a group founder; light blue-circle outlines indicate relatedness to the founder. The number above the line connecting two circles indicates the number of locus variants of seven loci that determine the ST types of the two circles. Colored pie charts indicate bacteriocin types and their proportion within each ST.

A total of 180 KpSC genomes were included in this study, comprising 170 isolates with the HMV phenotype and 10 isolates with the non-HMV phenotype (12). Subspecies analysis showed that these KpSC members included 165 (91.7%) K. pneumoniae strains, 1 (0.6%) Klebsiella quasipneumoniae subsp. *quasipneumoniae* strain, 7 (3.9%) Klebsiella quasipneumoniae subsp. *similipneumoniae* strains, and 7 (3.9%) Klebsiella variicola subsp. *variicola* strains (Fig. 1).

### Prevalence of bacteriocin genes.

Based on genome analysis, six types of bacteriocins, including microcin E492, microcin S-like, microcin B17, cloacin-like, klebicin B-like, and klebicin C-like, were identified in 32.8% (59/180) of the isolates. Bacteriocins whose amino acid sequences were not 100% identical to the published sequences were named with the suffix -like, for example, microcin S-like (see “Variations in bacteriocins” below). Bacteriocin genes were detected from 32.35% (55/170) of HMV isolates and 40% (4/10) of non-HMV isolates.

Among the bacteriocin gene-positive population, most strains (26/59 [44.1%]) possessed the microcin E492 gene (Table 1). Klebicin B-like and cloacin-like accounted for the second and third highest proportions, with 28.8% and 22.0%, respectively. The remaining bacteriocin genes were detected at relatively low rates: 5.1% (3/59) for microcin S-like and klebicin C-like and only 1.7% (1/59) for microcin B17.

**TABLE 1:** Prevalence of bacteriocins among 180 KpSC strains and number of variants of each bacteriocin

Bacteriocin	No. of strains	Prevalence (%) among:		No. of variants (amino acid level)
		Studied population (*n* = 180)	Bacteriocin-carrying strains (*n* = 59)	
Microcin E492	26	14.4	44.1	1
Microcin S-like bacteriocin	3	1.7	5.1	2
Microcin S-a	2			
Microcin S-b	1			
Microcin B17	1	0.6	1.7	1
Cloacin-like bacteriocin	13	7.2	22.0	5
Cloacin a	5			
Cloacin b	3			
Cloacin c	2			
Cloacin d	1			
Cloacin e	2			
Klebicin B-like bacteriocin	17	9.4	28.8	2
Klebicin B-a	2			
Klebicin B-b	1			
Klebicin B-X (klebicin B gene was disrupted)	14			
Klebicin C-like bacteriocin	3	1.7	5.1	2
Klebicin C-a	1			
Klebicin C-b	2			

Overall, among 59 isolates, 56 (94.9%), 2 (3.4%), and 1 (1.7%) carried single, double, and triple bacteriocins, respectively (Table 2). Klebicin C-like bacteriocin was detected as a single bacteriocin only. Microcin E492, klebicin B-like, and cloacin-like were frequently detected as single bacteriocins, except in the case of one strain that carried both microcin E492 and microcin B17 and one strain that carried both cloacin-like and klebicin B-like. Only one strain simultaneously carried 3 types of bacteriocins, microcin E492, microcin S-like, and klebicin B-like (Table 2).

**TABLE 2:** Number of isolates that carried one, two, or three bacteriocins

Bacteriocin(s)	No. (%) carrying:		
	Single bacteriocin	Double bacteriocins	Triple bacteriocins
Microcin E492	24 (40.7)		
Microcin S-like	2 (3.4)		
Cloacin-like	12 (20.3)		
Klebicin B-like	15 (25.4)		
Klebicin C-like	3 (5.1)		
Microcin E492 + microcin B17		1 (1.7)	
Cloacin-like + klebicin B-like		1 (1.7)	
Microcin E492 + microcin S-like + klebicin B-like			1 (1.7)
Total	56 (94.9)	2 (3.4)	1 (1.7)

### Multilocus sequence typing (MLST) and phylogenetic analysis.

Bacteriocin genes were detected in 33.3%, 0%, 28.6%, and 28.6% of K. pneumoniae, *K. quasipneumoniae* subsp. *similipneumoniae*, *K. quasipneumoniae* subsp. *similipneumoniae*, and *K. variicola* subsp. *variicola*, respectively. A total of 72 sequence types (STs) were identified, and at least one bacteriocin type was detected in 24/72 STs (33.3%) (Fig. 1). The dominant STs included ST23 (27/180 strains [15%]), ST65 (19/180 strains [10.6%]), ST86 (15/180 strains [8.3%]), ST268 (9/180 strains [5.0%]), and ST37 (9/180 strains [5.0%]). Among these five prominent STs, ST23 and ST37 strains were frequently positive for microcin E492 (24/27 strains [88.9%]) and cloacin-like/klebicin B-like (7/9 strains [77.8%]), respectively, while ST65, ST86, and ST268 were negative for known bacteriocins (Fig. 1). Bacteriocins were also detected in some minor STs, such as ST36 (3/4 strains [75%]), ST919 (2/4 strains [50%]), ST11, ST1061, and ST5134 (2/2 strains of each ST [100%]), and other STs, as shown in Fig. 1.

**FIG 2:**
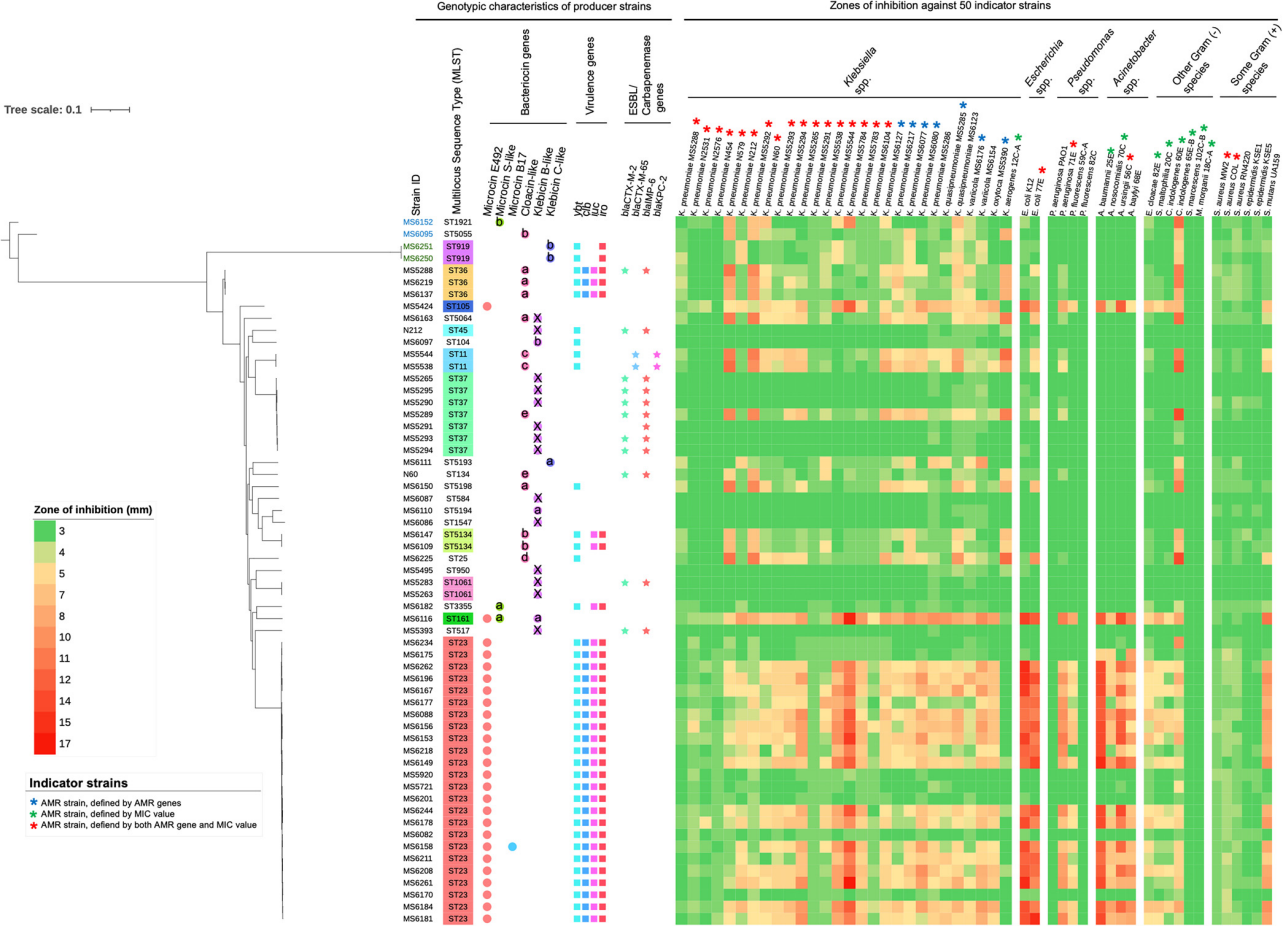
Phylogenetic tree of 59 bacteriocin-carrying KpSC strains, distribution of bacteriocin types, and their antibacterial activity. The black, green, and blue colors of the strain identifier (ID) indicate K. pneumoniae, K. pneumoniae *variicola* subsp. *variicola*, and *K. quasipneumoniae* subsp. *similipneumoniae*, respectively. Genotypic characteristics, including the STs, bacteriocin genes, virulence profiles, and ESBL/carbapenemase profiles, are plotted next to the strain IDs. The characters a, b, c, d, and e indicate different variants of each bacteriocin type. The X symbol for klebicin B indicates a disrupted gene. Data of the diameters of growth inhibition zones against 50 indicator strains from soft-agar overlay assay are displayed as the heat map on the right. An inhibition zone of 3 mm means no antibacterial activity.

A phylogenetic tree was created based on whole-genome sequences (WGS) of these 59 isolates, and various types of information (ST types, bacteriocin types, virulence genes, and antimicrobial resistance [AMR] genes) were plotted next to each strain (Fig. 2). Strikingly, 92.3% (24/26) of the microcin E492-carrying strains belonged to ST23. All of these ST23 isolates were positive for four virulence genes, including yersiniabactin (*ybt*), colibactin (*clb*), aerobactin (*iuc*), and salmochelin (*iro*) genes (Fig. 2). Interestingly, the other two E492-carrying isolates, MS5424 and MS6116, belonged to other miscellaneous STs (ST105 and ST161, respectively) and were negative for all four virulence genes. Within ST37, 85.7% (6/7) of the isolates carried the klebicin B-like gene, while only 1 strain possessed the cloacin-like gene (MS5289). These seven isolates were negative for virulence genes but carried extended-spectrum beta-lactamase (ESBL) and carbapenemase genes (*bla*_CTX-M-2_ and *bla*_IMP-6_). ST36 (3 strains) and ST5134 (2 strains) carried cloacin-like and virulence genes. ST11 (2 strains) was positive for cloacin-like, ESBL, and carbapenemase (*bla*_CTX-M-65_ and *bla*_KPC-2_) genes. Klebicin C-like bacteriocin was present in one K. pneumoniae isolate (MS6111 [ST5193]) and two *K. variicola* isolates (MS6250 and MS6251 [both ST919]). Microcin S-like was identified from two K. pneumoniae isolates (MS6116 [ST161] and MS6182 [ST3355]) and one *K. quasipneumoniae* isolate (MS6152 [ST1921]). Between the two microcin S-like-positive K. pneumoniae strains, one (MS6182) carried microcin S-like as a single bacteriocin, while the other (MS6116) carried microcin S-like together with microcin E492. Microcin B17 was found in only one K. pneumoniae isolate (MS6158), which belonged to ST23 and simultaneously carried microcin E492.

### Variations in bacteriocins.

At the amino acid level, cloacins exhibited the most varied structure, with five variants, while the other bacteriocins showed only one (microcin E492 and microcin B17) or two (microcin S-like, klebicin B-like, and klebicin C-like) variants (Table 1).

**FIG 3:**
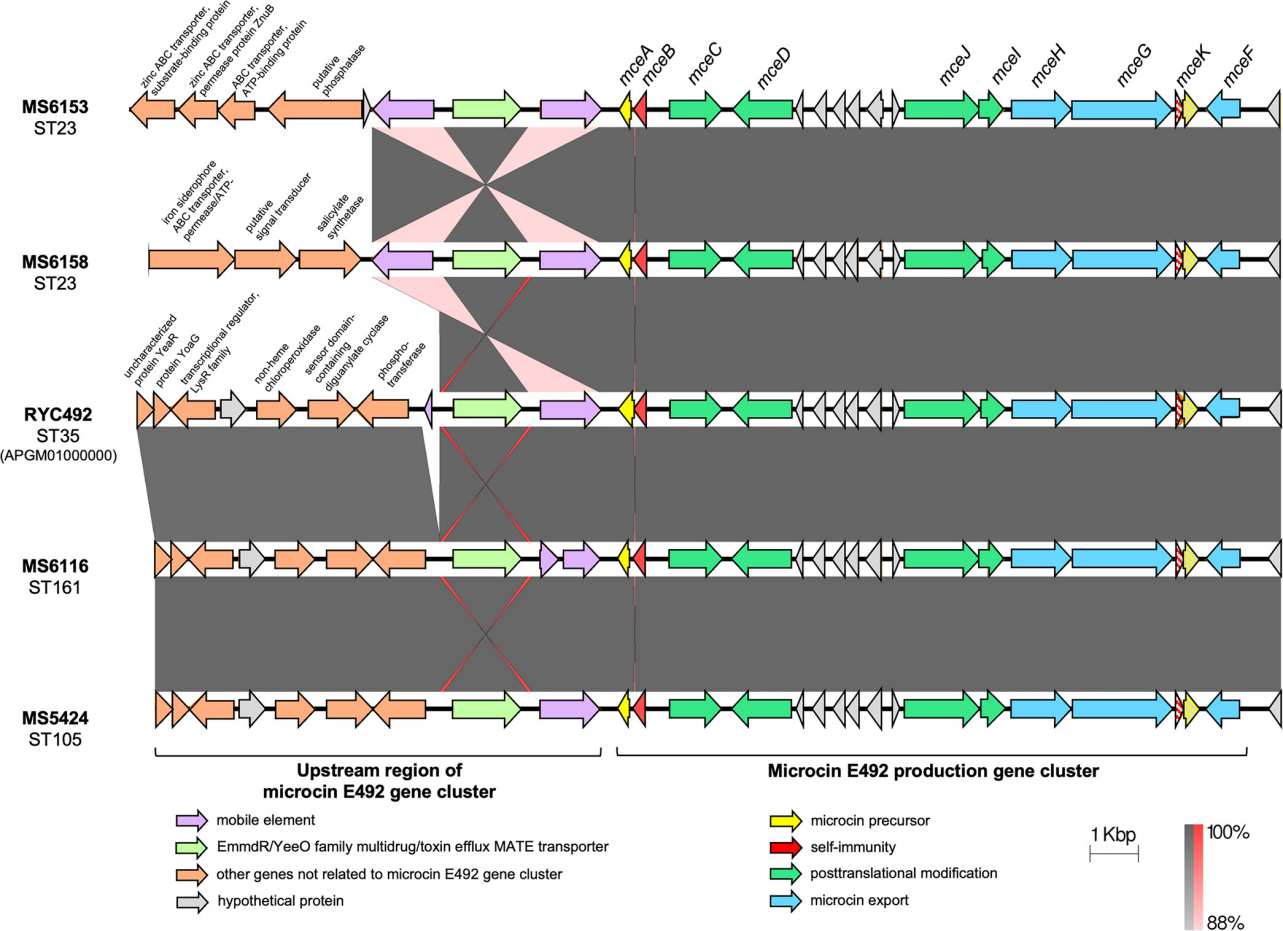
Alignment of the microcin E492 production gene cluster and its upstream region. The microcin E492 gene cluster (from *mceA* to *mceF*) and about 9.5 to 10 kbp upstream of *mceA* were extracted from two ST23 isolates (MS6153 and MS6158), one ST161 isolate (MS6116), and one ST105 isolate (MS5424) and were compared with that of strain RYC492 (GenBank accession number APGM01000000). The gray scale indicates the percent sequence identity, and the red scale indicates the percent sequence identity of inverted sequences. Genes are presented as arrows, with the orientations referring to direction of gene transcription. Truncated genes are displayed as striped arrows.

Microcin E492 amino acid sequences were identical among all strains and were identical to that of strain RYC492 (GenBank accession numbers AF063590 and APGM01000000). The microcin E492 gene clusters and around 9.5 to 10 kbp upstream of *mceA*, which encodes the microcin E492 precursor, were extracted from two representative ST23 isolates (MS6153 and MS6158), one ST105 isolate (MS5424), and one ST161 isolate (MS6116). The alignment of these sequences with the respective sequence from RYC492 (GenBank accession number APGM01000000) is displayed in Fig. 3. The results showed that the five strains carried similar structures with the microcin E492 gene cluster, while the upstream regions varied between the ST23 and non-ST23 strains. The two ST23 isolates exhibited similar 5-kbp regions upstream of *mceA*, with the presence of two mobile elements flanking one EmmdR/YeeO family multidrug/toxin efflux MATE transporter gene. However, the further 5-kbp regions upstream of the mobile elements were totally different between MS6153 and MS6158. In contrast, the remaining three strains, which belonged to three distinct STs (ST35, ST161, and ST105), displayed high-homology structures within the whole 10-kbp region upstream of *mceA*. The microcin E492 loci from these three non-ST23 isolates were uniformly inserted into the DNA region next to the phosphotransferase gene (Fig. 3).

**FIG 4:**
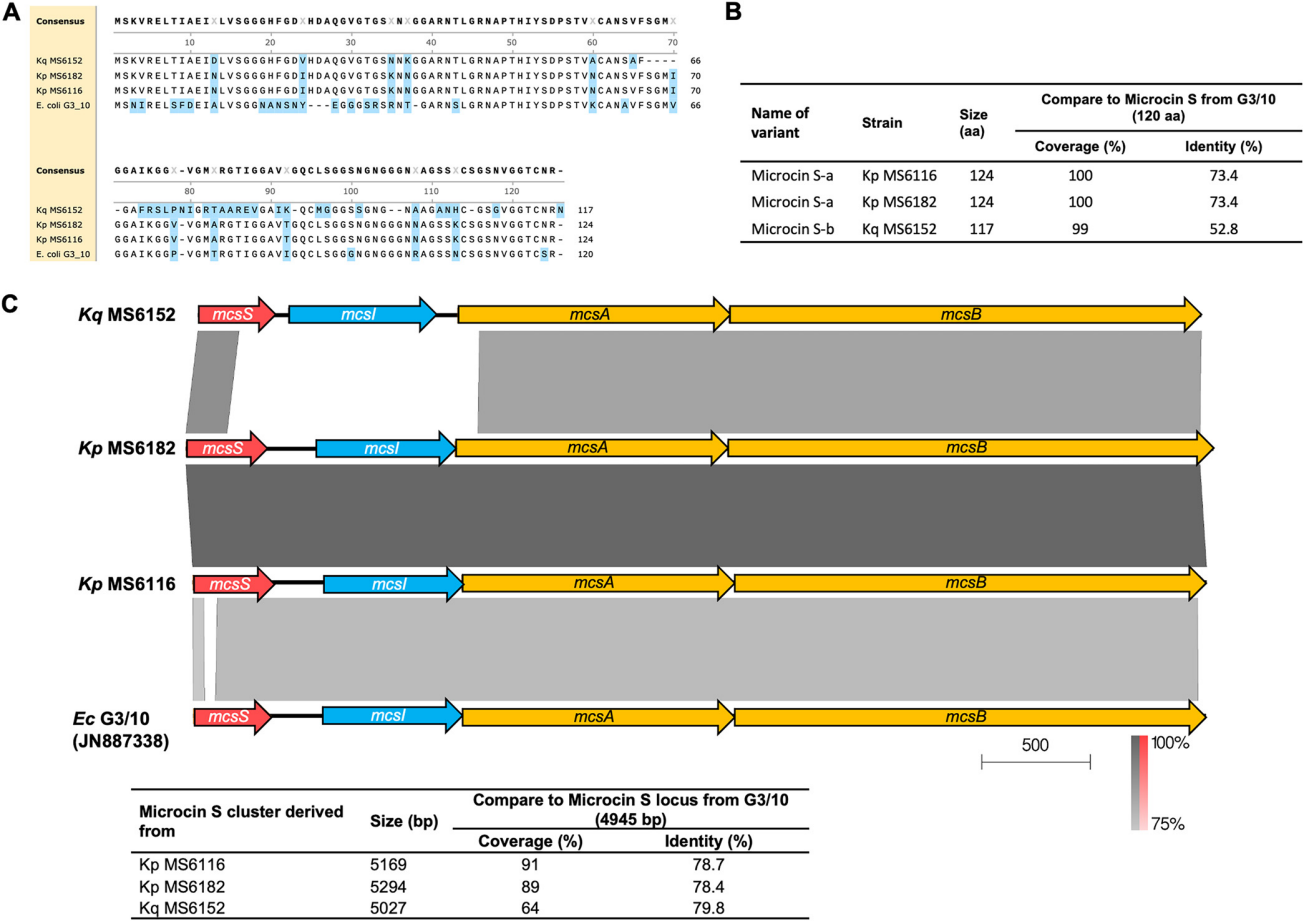
Comparison of microcin S-like bacteriocins. (A) Amino acid alignment of microcin S-like bacteriocins from two K. pneumoniae strains (MS6116 and MS6182) and one *K. quasipneumoniae* strain (MS6152) against the reference microcin S gene from E. coli G3/10 (GenBank accession number JN887338). (B) Percent coverage and percent identity of amino acid (aa) sequences of the a and b variants of microcin S compared with that from E. coli G3/10. (C) Microcin S gene cluster comparison among the three strains and the E. coli G3/10 strain. The gray scale indicates the percent sequence identity, and the red scale indicates the percent sequence identity of inverted sequences. Genes are presented as arrows, with the orientations referring to direction of gene transcription.

Microcin S-like bacteriocin had two variants, which differed in the amino acid lengths (124 and 117 amino acid residues, respectively) and shared 65% (80/124) identity (Fig. 4A). The variant microcin S-a was derived from two K. pneumoniae strains (MS6116 and MS6182), and microcin S-b was derived from one *K. quasipneumoniae* strain (MS6152), sharing 73.4% and 52.8% identity with McsS from E. coli G3/10 (13), respectively (Fig. 4B). We next compared the microcin S gene cluster of our three strains with that of E. coli G3/10 (GenBank accession number JN887338). The results indicated that the genetic component of the microcin S locus was conserved, including the microcin S precursor (*mcsS*), a gene encoding the self-immunity protein (*mcsI*), and two genes involved in the microcin export system (*mcsA* and *mcsB*) (Fig. 4C). At the nucleotide level, the clusters from two K. pneumoniae strains, MS6116 and MS6182, were nearly identical and conferred approximately 90% coverage and 78% identity to that from E. coli G3/10. Meanwhile, the cluster from *K. quasipneumoniae* MS6152 differed from the others at the *mcsI* gene and approximately 250 bp upstream and downstream of *mcsI*, which included a part of *mcsS* and *mcsA* (Fig. 4C).

**FIG 5:**
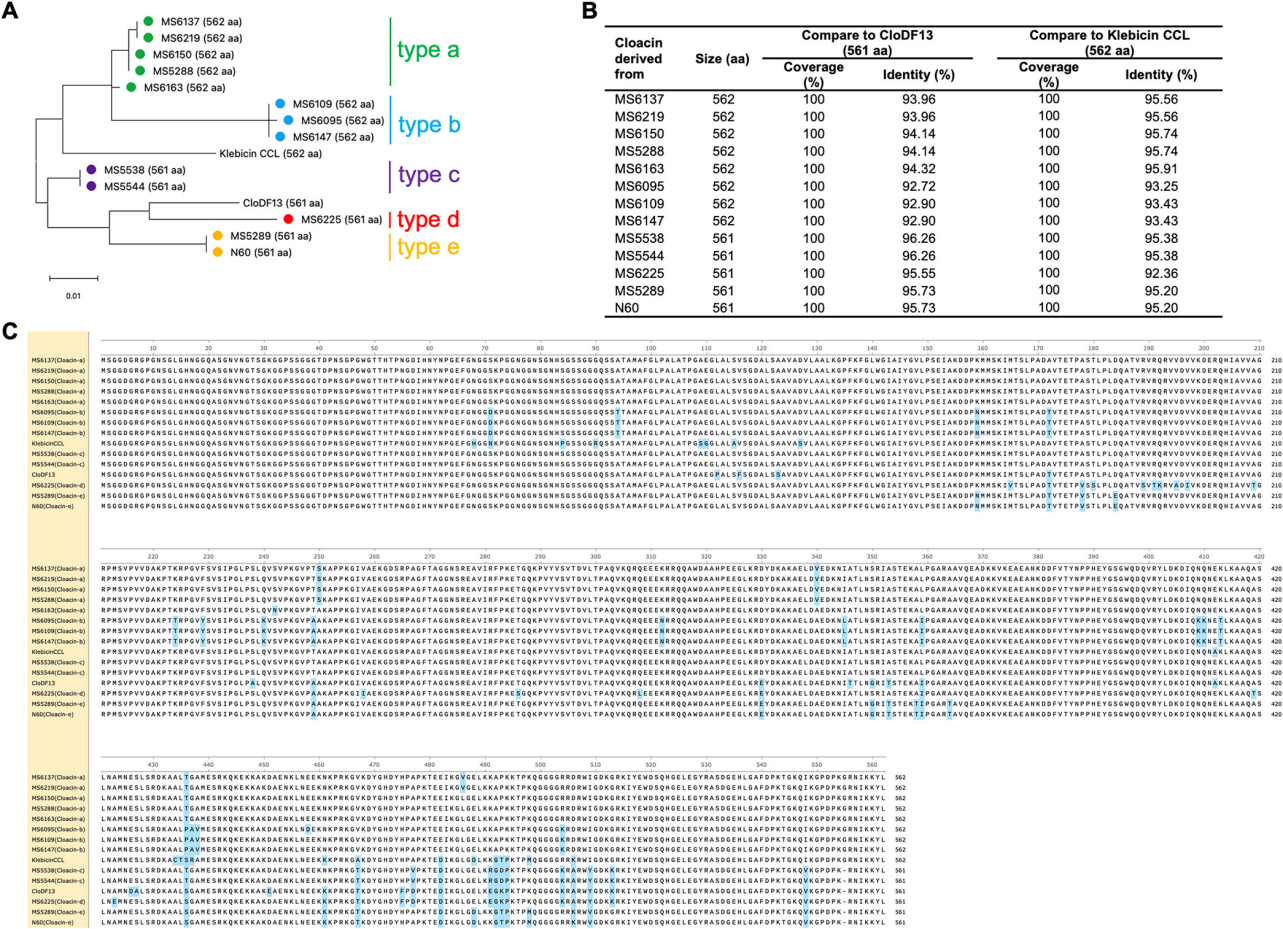
Comparison of cloacin-like amino acid sequences. (A) Phylogenetic tree analysis of cloacin-like amino acid sequences derived from 13 KpSC isolates and two references, CloDF13 (GenBank accession number X04466) and klebicin CCL (GenBank accession number AF190857). (B) Percent coverage and percent identity of the amino acid sequence of all variants of cloacin in comparison with CloDF13 and klebicin CCL. (C) Amino acid alignment of different cloacin variants.

**FIG 6:**
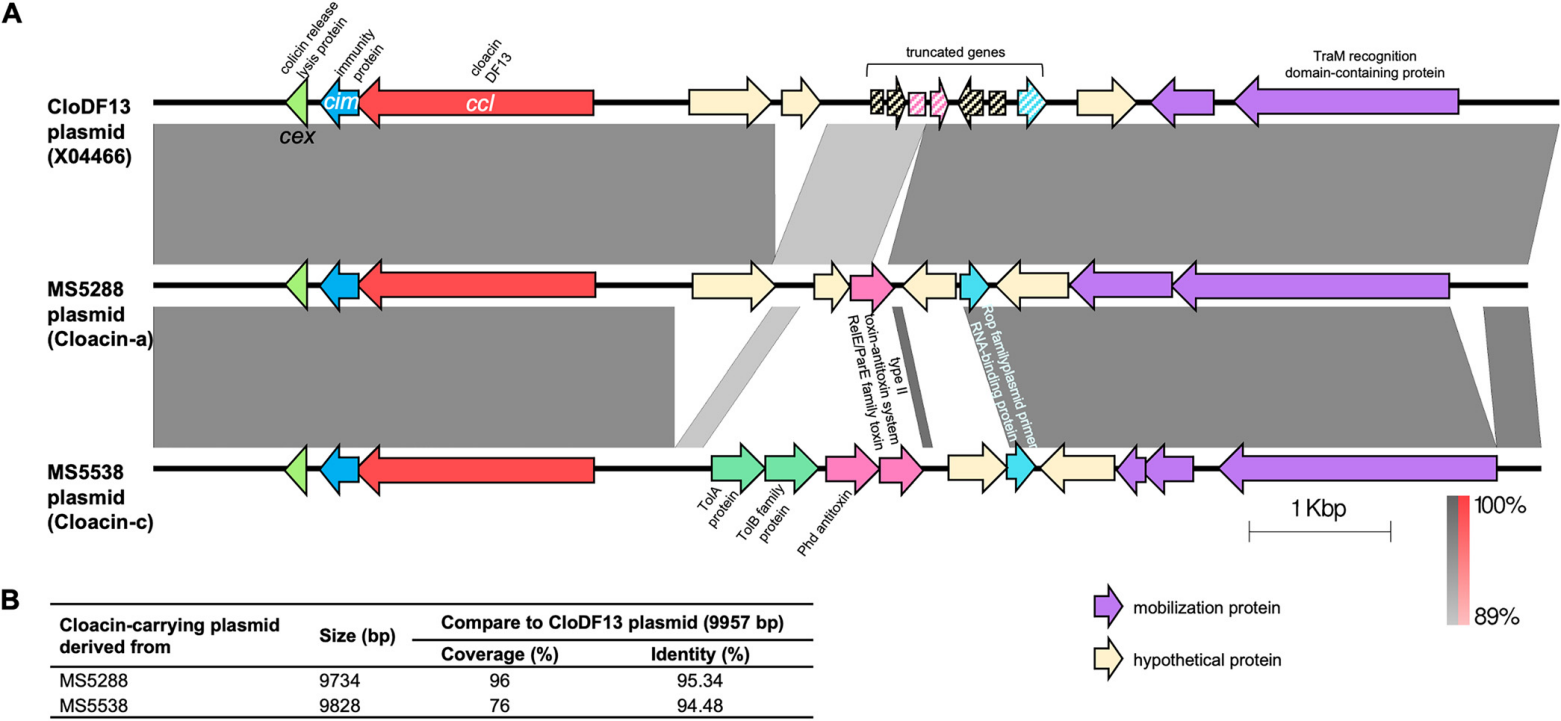
Alignment of cloacin-like-carrying plasmids. (A) The complete sequences of cloacin-like-carrying plasmids from MS5288 and MS5538 were selected and aligned with the CloDF13 plasmid (GenBank accession number X04466). The gray scale indicates the percent sequence identity, and the red scale indicates the percent sequence identity of inverted sequences. Genes are presented as arrows, with the orientations referring to direction of gene transcription. Truncated genes are displayed as striped arrows. (B) Percent coverage and percent identity of nucleotide sequence between the cloacin-like-carrying plasmids from MS5288 and MS5538 in comparison with the CloDF13 plasmid.

Cloacins were categorized into 5 types (a, b, c, d, and e), with 92.90% to 96.26% identity to the CloDF13 sequence and 92.36% to 95.91% identity to klebicin CCL (Fig. 5A and B). Cloacin types a and b carried 562 amino acid residues and were closer to klebicin CCL, while types c, d, and e carried 561 amino acid residues and were closer to cloacin DF13. Amino acid sequence alignment showed that the N-terminal sequences were quite conserved, while more variations were observed within the central and C-terminal regions (Fig. 5C). The cloacin-carrying plasmid comparison among the complete sequences of the bacteriocinogenic plasmid CloDF13 (GenBank accession number X04466) and those derived from our two representative strains, MS5288 (cloacin a) and MS5538 (cloacin c), is displayed in Fig. 6. All three plasmids carried a conserved structure of the bacteriocin-related locus, including the cloacin bacteriocin gene, the immunity protein, the colicin release lysis protein, and a region of TraM recognition domain-containing protein and some other proteins that assist in plasmid mobilization. However, the middle regions of these three plasmids were variable (Fig. 6A). This region of the plasmids from MS5288 and MS5538 carried genes coding for toxin-antitoxin system RelE/ParE family toxin and Rop family plasmid primer RNA-binding protein; however, the respective genes in the CloDF13 plasmid were truncated. In addition, the TolA protein and TolB family proteins were present in the MS5538-derived plasmid but were not found in the other two plasmids. The nucleotide sequence of the plasmid from MS5288 shared higher homology with the CloDF13 plasmid (96% coverage and 95.34% identity) than the plasmid from MS5538 did (76% coverage and 94.48% identity) (Fig. 6B).

**FIG 7:**
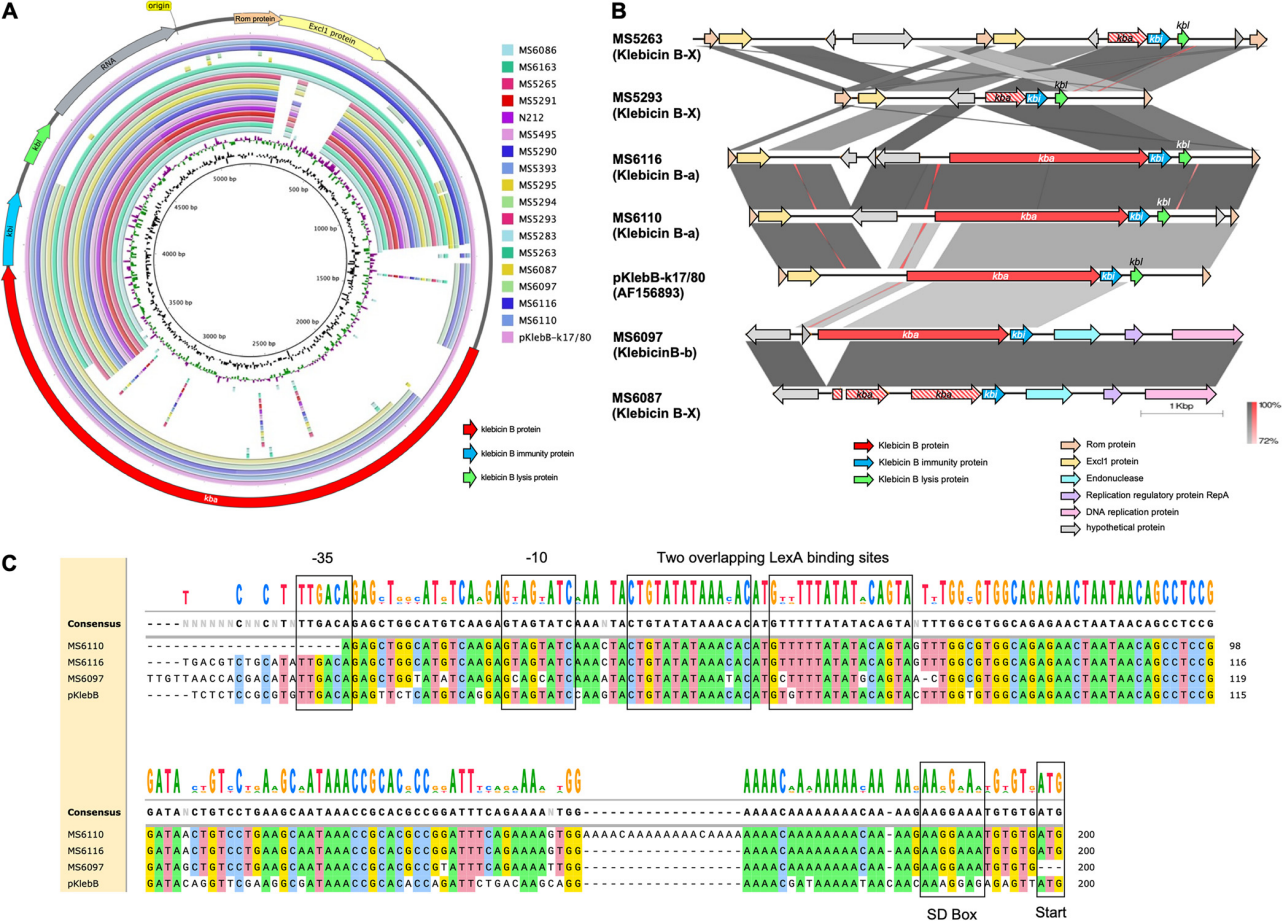
Comparison of klebicin B-like gene clusters. (A) BLAST comparison of the klebicin B-like gene cluster from 17 isolates in this study using the public plasmid sequence pKlebB-k17/80 (GenBank accession number AF156893) as the reference. The two innermost circles represent the GC content (black) and GC skew (purple/green). The color key provides the names of 17 isolates, arranged from inner to outer colored rings. The outermost circle displays previously reported coding sequences of pKlebB-k17/80. (B) Linear alignment of klebicin B-like gene cluster from 5 representative isolates and pKlebB-k17/80 (GenBank accession number AF156893). The gray scale indicates the percent sequence identity, and the red scale indicates the percent sequence identity of inverted sequences. Genes are presented as arrows, with the orientations referring to direction of gene transcription. Truncated genes are displayed as striped arrows. (C) Alignment of the promoter regions of *kba* from MS6110, MS6116, MS6097, and pKlebB-k17/80.

Klebicin B-like bacteriocin was detected in 9.4% (17/59) of the isolates; however, 82.4% (14/17 strains) carried a disrupted bacteriocin gene and hence may not produce a functional bacteriocin (Table 1). Figure 7 displays the comparison of klebicin B-like gene clusters from our isolates using the pKlebB-k17/80 (GenBank accession number AF156893) as a reference. The data showed that 13/17 isolates carried a disrupted *kba* gene, which covered only about one-fourth of the length of that of pKlebB-k17/80 (Fig. 7A and B). The *kba* genes from MS6110, MS6116, MS6097, and MS6087 covered approximately 70 to 80% of the length of that from pKlebB-k17/80. Nevertheless, the Kba protein of MS6087 was truncated into several fragments due to the introduction of mutations and thus may not produce a functional bacteriocin. Therefore, only three isolates (MS6110, MS6116, and MS6097) were regarded as carrying an intact *kba*, although their nucleotide sequences showed low homology with that of pKlebB-k17/80 (Fig. 7B). The *kba* sequences of MS6110 and MS6116 were identical and were designated the klebicin B-a variant, the *kba* from MS6097 was named the klebicin B-b variant, and the disrupted or truncated *kba* was marked as the klebicin X variant. At the amino acid level, the klebicin B-a and -b variants displayed 99% coverage and 66.37% and 67.66% identity with klebicin B from pKlebB-k17/80, respectively (see Fig. S1 in the supplemental material). Interestingly, all 17 strains carried a complete *kbi* immunity gene regardless of the disruption or truncation of *kba*. The *kbl* lysis gene was observed in most of the strains except MS6097 and MS6087, although MS6097 carried an intact *kba* (Fig. 7A and B). Comparison of the promoter regions upstream of *kba* of the three isolates exhibited several mutations at the −35 sequences, −10 sequences, LexA binding sites, and Shine-Dalgarno (SD) box compared to pKlebB-k17/80 (Fig. 7C).

**FIG 8:**
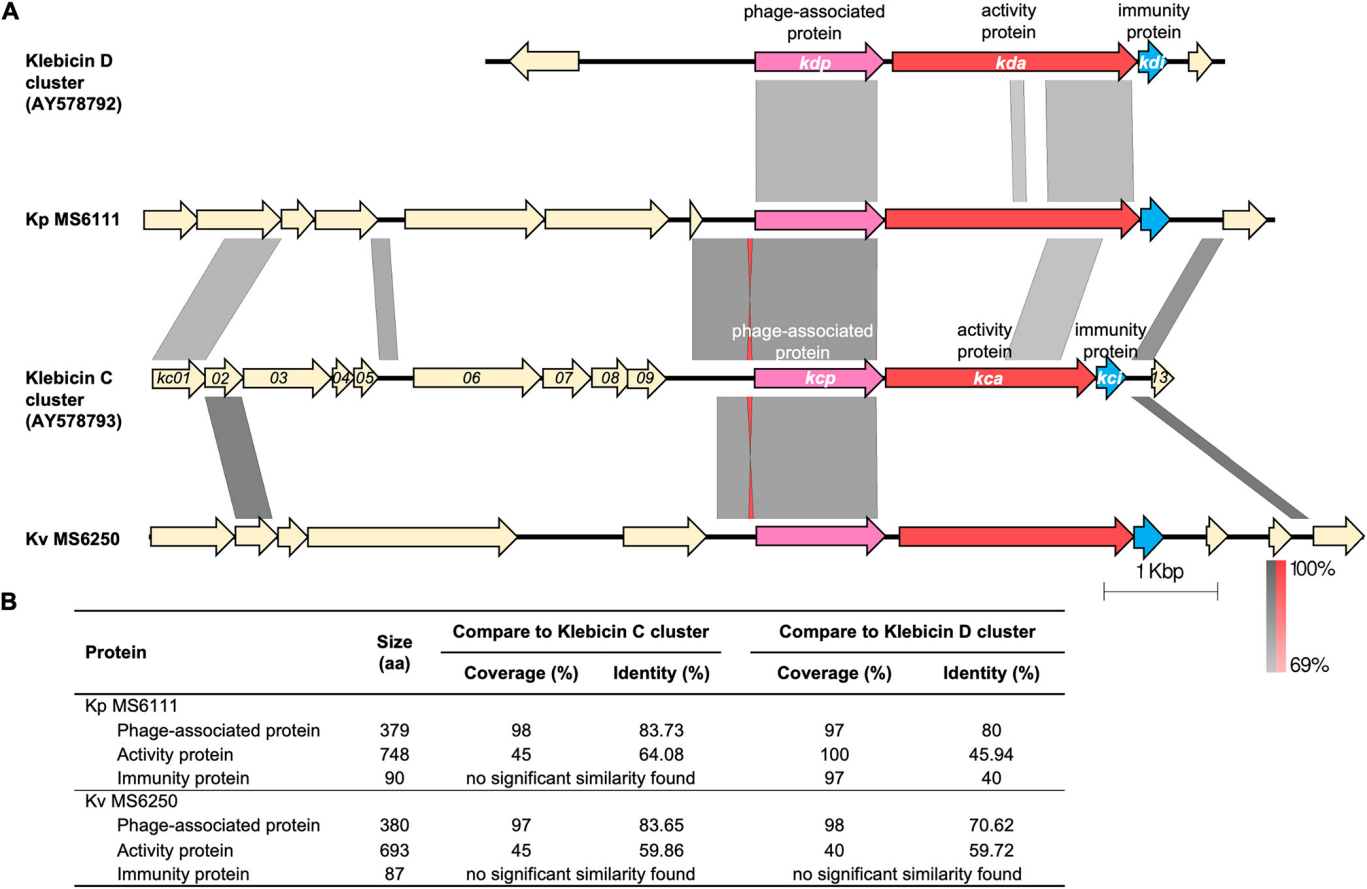
Comparison of the klebicin C-like gene clusters. (A) The klebicin C-like gene clusters from MS6111 and MS6250 were selected and aligned with the klebicin C cluster (GenBank accession number AY578793) and klebicin D cluster (GenBank accession number AY578792). The gray scale indicates the percent sequence identity, and the red scale indicates the percent sequence identity of inverted sequences. Genes are presented as arrows, with the orientations referring to direction of gene transcription. (B) Percent coverage and percent identity of amino acid sequences of the phage-associated protein, the activity protein, and the immunity protein between MS6111 or MS6250 and klebicin C or klebicin D.

Klebicin C-like bacteriocin was found in three strains with two variations, namely, klebicin C-a (in K. pneumoniae MS6111) and klebicin C-b (in *K. variicola* MS6250 and MS6251) (Table 1 and Fig. 1). The gene structure comparison of the two klebicin C-like gene clusters and the klebicin C (GenBank accession number AY578793) and klebicin D (GenBank accession number AY578792) clusters is shown in Fig. 8. Although these clusters consisted of similar components, including a phage-associated gene (*kcp/kdp*-like gene), an activity gene (*kca/kda*-like gene), and an immunity gene (*kci*/*kdi*-like gene), the activity and immunity genes from MS6111 and MS6250 showed limited or no sequence similarity with those of the klebicin C or klebicin D clusters (Fig. 8A). The phage-associated protein sequences from MS6111 and MS6250 were closer to Kcp (98% and 97% coverage and 83.73% and 83.65% identity, respectively) than to Kdp (97% and 98% coverage and 80% and 70.62% identity, respectively). However, the activity protein displayed significantly lower similarity to both Kca and Kda (≤64.08% identity), and almost no significant similarity was found between the immunity proteins of MS6111/MS6250 and Kci/Kdi (Fig. 8B).

### Antibacterial activity.

**(i) General antibacterial activity against Gram-negative bacteria and some Gram-positive bacteria.** The antibacterial activity of different bacteriocin types was tested against a variety of indicator species with both drug-susceptible and drug-resistant strains belonging to Klebsiella spp., Escherichia spp., Pseudomonas spp., Acinetobacter spp., other Gram-negative species, and some Gram-positive species (Table S1). The zones of inhibition against 50 indicator strains are displayed in the right panel of Fig. 2. In general, the microcin E492-carrying strains conferred a higher inhibitory effect and a wider spectrum of activity than the strains with other bacteriocin types. Microcin S-like displayed activity against some K. pneumoniae and Chryseobacterium indologenes indicators. Cloacin-like-positive strains showed activities against certain indicator strains, mainly Klebsiella spp., E. coli, and *C. indologenes*, while klebicin B-like-positive strains displayed almost no inhibitory effect against the tested indicators. Klebicin C-like exhibited mild activity only against some K. pneumoniae, *K. quasipneumoniae*, and *K. variicola* strains.

**FIG 9:**
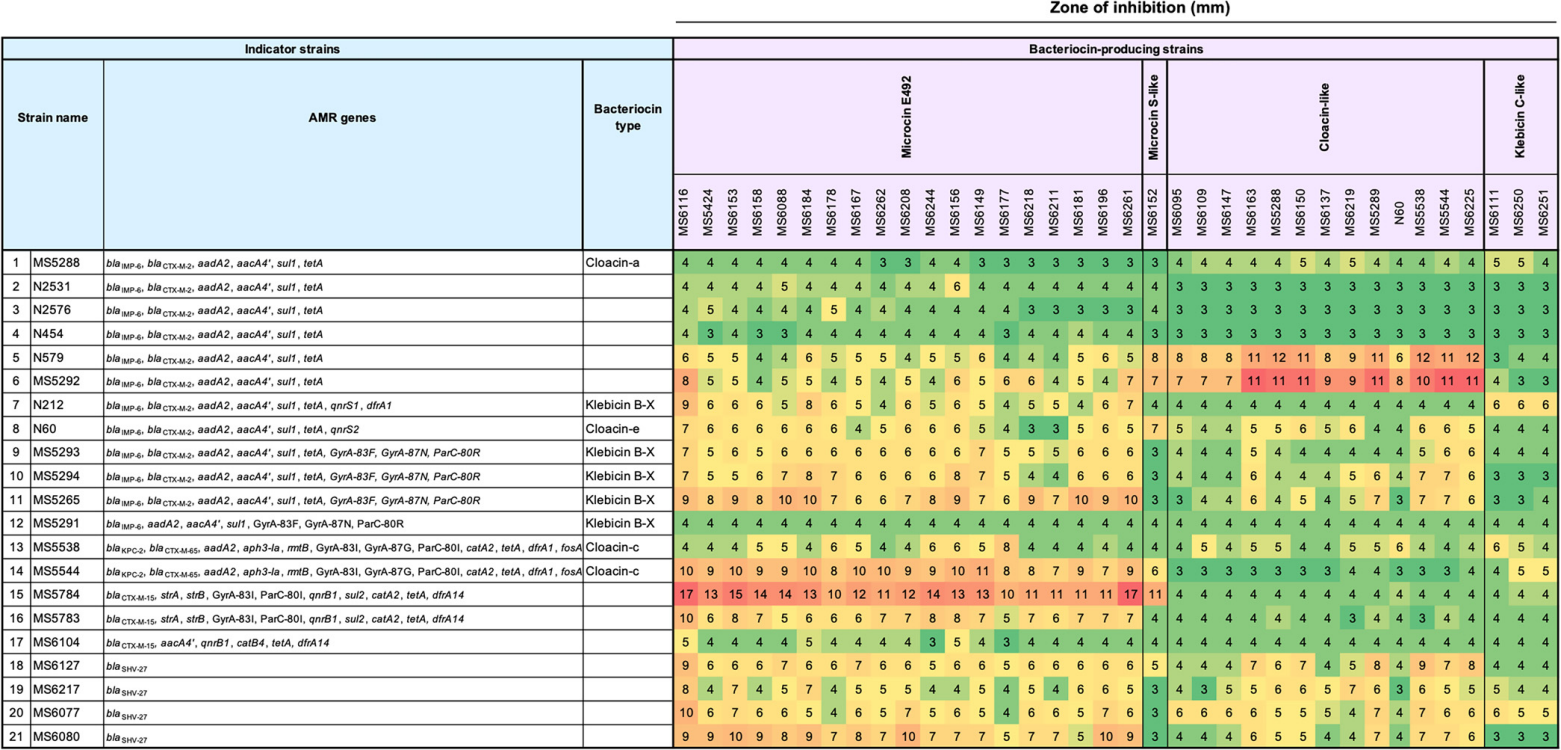
Inhibitory activities of different bacteriocins against 21 AMR K. pneumoniae strains. The antimicrobial activity of 36 strains carrying microcin E492, microcin S-like, cloacin-like, and klebicin C-like bacteriocin against 21 AMR K. pneumoniae indicator isolates was investigated. Diameters of growth inhibition zones from soft-agar overlay assay are displayed as the numbers and heat map on the right. An inhibition zone of 3 mm means no antibacterial activity. The AMR genes and bacteriocin type of each indicator strain are also displayed.

**(ii) Inhibitory activities against 21 AMR K. pneumoniae strains.** The antibacterial activities of 59 bacteriocin-carrying isolates was tested against 21 AMR K. pneumoniae strains isolated previously (12). The results indicated that some ESBL- and/or carbapenemase-producing strains, which showed resistance to multiple beta-lactams, carbapenems, and other antibiotics, had intermediate or high susceptibility to certain bacteriocins, except for five AMR isolates that displayed resistance to all bacteriocins (MS5288, N2531, N2576, N454, and MS5291) (Fig. 2 and 9). Microcin E492 generally had stronger activity (the zone of inhibition in some cases was over 10 mm) against a larger number (15/21 strains [71.4%]) of AMR K. pneumoniae strains than other bacteriocins. Notably, some ESBL- and/or carbapenemase-producing K. pneumoniae isolates, e.g., a *bla*_KPC-2_- and *bla*_CTX-M-65_-carrying strain (MS5544), a *bla*_IMP-6_- and *bla*_CTX-M-2_-carrying strain (MS5265), and a *bla*_CTX-M-15_-carrying strain (MS5784), displayed high susceptibility to microcin E492 (Fig. 9). Cloacin-like substantially inhibited two strains (N579 and MS5292, both of which carried *bla*_IMP-6_ and *bla*_CTX-M-2_) while showing an intermediate to low effect against the other indicators.

**FIG 10:**
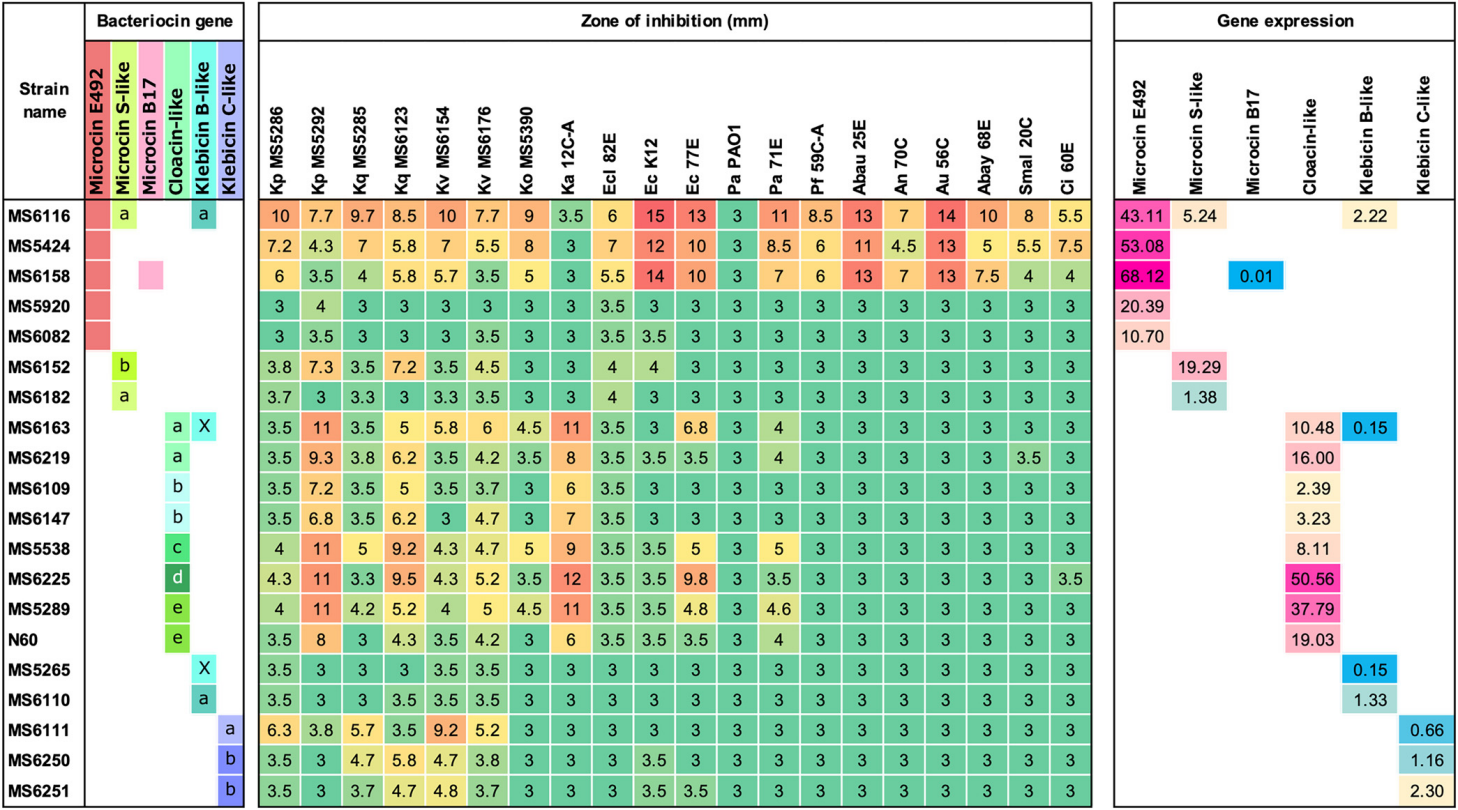
Antibacterial activities and bacteriocin gene expressions of representative strains. RNA extraction and quantitative PCR were performed on representative isolates of each bacteriocin type to observe the correlation between the zones of inhibition and gene expression. Diameters of growth inhibition zones from soft-agar overlay assay are displayed as the numbers and heat map on the left. An inhibition zone of 3 mm means no antibacterial activity. The gene expression of each bacteriocin gene relative to *recA* is displayed as a heat map on the right.

**(iii) Gene expression in correlation with antibacterial activity.** Representative strains with each group of bacteriocins were selected for measurement of gene expression in solid media by quantitative PCR (qPCR). The expression of different genes varied considerably (Fig. 10). Generally, microcin E492 and cloacin-like were expressed at high levels, while the expression of klebicin B17, klebicin B-like, and klebicin C-like was low. The expression of microcin E492 and microcin S-like was proportional to the antimicrobial effects. The low antibacterial activity of some microcin E492-carrying strains (MS5920 and MS6082) was due to the lower expression of the respective bacteriocin gene than that of MS5424. MS6182 displayed low antimicrobial activity due to the low expression of microcin S-like compared to that of MS6152. The expression of cloacin-like varied among different strains and with different variants of cloacin. Notably, cloacin type d in MS6225 was expressed at a remarkably high level, which may explain the high antibacterial activity against various species compared to the other cloacin-like-carrying strains. Between the two cloacin type e strains (MS5289 and N60), the antibacterial activity was correlated with the expression level of the cloacin-like gene. Klebicin B-like displayed low expression, and the klebicin B-like-single bacteriocin strains showed almost no inhibitory activity. The two isolates carrying the klebicin B-a variant (MS6116 and MS6110) exhibited a little higher expression of *kba* than that of the klebicin B-X-carrying strains (MS6163 and MS5265).

**FIG 11:**
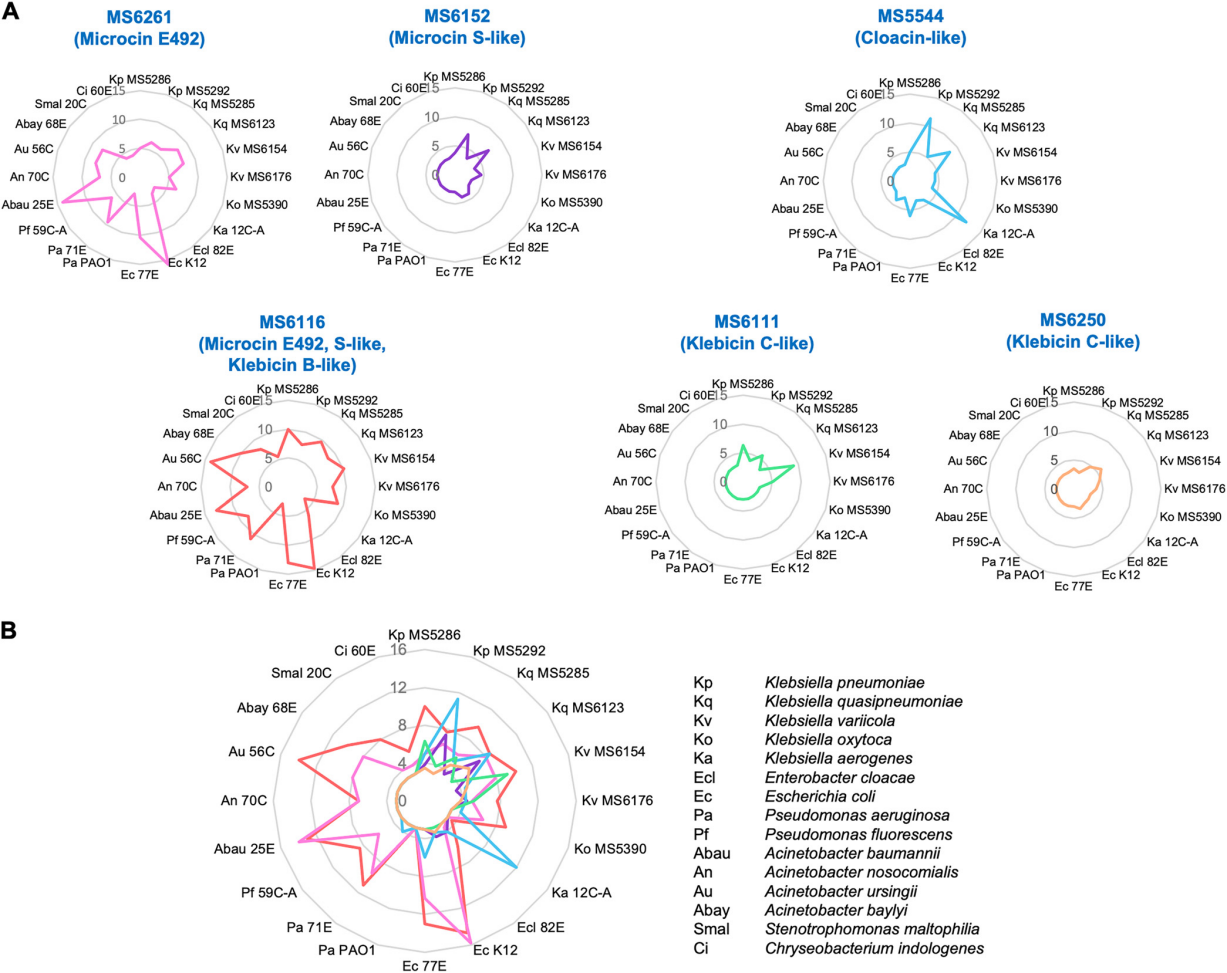
Antibacterial pattern of each bacteriocin type. The diameters of growth inhibition zones (in millimeters) of representative strains of each bacteriocin type against multiple indicator species were plotted to observe their general antibacterial spectrum. An inhibition zone of 3 mm means no antibacterial activity. (A) The antibacterial pattern of each bacteriocin type is displayed separately. (B) Antibacterial patterns in panel A are merged to compare the activities of different bacteriocin types.

**(iv) Antimicrobial patterns of different bacteriocins.** The activities of representative producers with different bacteriocin types are plotted in Fig. 11. The shapes of the radar charts varied significantly, but generally three patterns could be observed. Microcin E492 (MS6261) was effective against E. coli K-12, ESBL-producing E. coli 77E, MDR Pseudomonas aeruginosa 71E, Acinetobacter baumannii 25E, and Acinetobacter ursingii 56C but showed less activity toward Klebsiella species. In contrast, the cloacin-like-positive strain (MS5544) showed high activity against some Klebsiella spp. and mild activity against ESBL-producing E. coli 77E but almost no effect against Pseudomonas or Acinetobacter species. Microcin S-like-carrying (MS6152) and klebicin C-like-carrying (MS6250 and MS6111) strains tended to have mild inhibitory effects against some Klebsiella spp. but almost no activity against the other species. Interestingly, MS6116, which was positive for both microcin E492 and microcin S-like, displayed the combined spectra of activities that were seen in MS6261 (microcin E492 only) and MS6152 (microcin S-like bacteriocin only).

**FIG 12:**
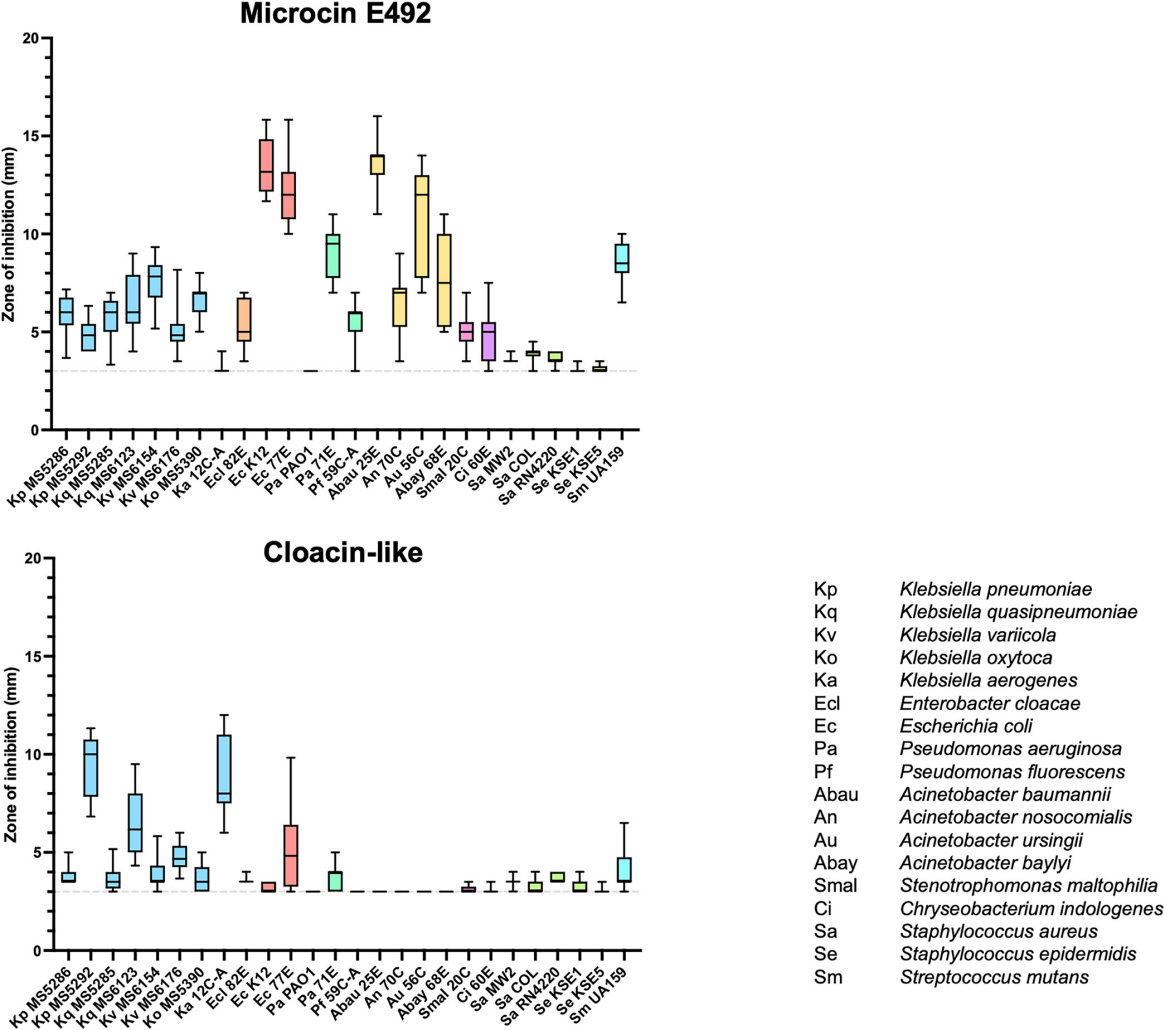
Antibacterial activity of the microcin E492-carrying group and the cloacin-like-carrying group. The mean value and distribution of diameters of growth inhibition zones of microcin E492-carrying strains and cloacin-like-carrying strains against each indicator strain were calculated and plotted. Different colors indicate different bacterial families. An inhibition zone of 3 mm means no antibacterial activity.

**(v) Comparison of antibacterial activities between the microcin E492-carrying group and the cloacin-carrying group.** The high prevalence of microcin E492 (44.1%) and cloacin (22.0%) with opposite spectra of activity prompted us to further investigate their median inhibitory effects against each indicator. The producers with no activity against all tested indicators were considered to have imperfect bacteriocin production and were eliminated. The mean zone of inhibition from all producers of the same bacteriocin against each indicator was then calculated and plotted in Fig. 12. Overall, strains producing microcin E492 had a stronger and broader spectrum of activity than the cloacin group. Microcin E492 strains displayed intermediate activity against the tested Klebsiella strains, E. cloacae 82E, MDR P. aeruginosa 71E, Pseudomonas fluorescens 59C-A, Acinetobacter nosocomialis 70C, Acinetobacter baylyi 68E, Stenotrophomonas maltophilia 20C, and *C. indologenes* 60E and high activity against two E. coli strains (K-12 and ESBL-producing strain 77E) and some Acinetobacter spp. (AMR A. baumannii 25E and AMR *A. ursingii* 56C). Additionally, they also had intermediate activity against Gram-positive Streptococcus mutans UA159, although low or no activity was seen against S. aureus or Staphylococcus epidermidis. In contrast, the cloacin-like-producing group displayed a relatively narrow spectrum of activity, with intermediate activities against certain strains of Klebsiella (AMR K. pneumoniae MS5292, *K. quasipneumoniae* MS6123, and AMR Klebsiella aerogenes 12C-A) and ESBL-producing E. coli 77E. Cloacin-like also showed mild activity against S. mutans UA159, but the activity was weaker than that of the microcin E492 group.

## DISCUSSION

In this study, we performed bacteriocin identification based on WGS of 180 clinically isolated KpSC strains, including 170 HMV and 10 non-HMV isolates, and investigated the antimicrobial spectrum of the strains that were bacteriocin positive. We determined that 32.8% (59/180) of the strains carried at least one known bacteriocin. Six types of bacteriocins were identified, some of which carried several variants, such as microcin S-like, cloacin-like, klebicin B-like, and klebicin C-like bacteriocins (Table 1). Microcin E492, klebicin B-like bacteriocin, and cloacin-like bacteriocin accounted for the three most prevalent bacteriocins among the 180 isolates (Table 1). Bacteriocin genes were detected in 33.3% (24/72) of the MLSTs in our collection. Certain specific STs carried bacteriocins with rates over 50%, while some other STs were negative for all bacteriocin genes (Fig. 1), suggesting the ecological role of bacteriocin in different STs. To the best of our knowledge, this is the first comprehensive report about the prevalence and distribution of bacteriocins (klebocins) in the KpSC population.

We also tested the antibacterial activities of 59 bacteriocin gene-positive strains against 50 clinically isolated indicators belonging to various species, including MDR strains. Generally, the microcin E492-carrying strains displayed a strong and broad spectrum of antibacterial activity, while the cloacin-like-carrying strains displayed activity against closely related species belonging to Klebsiella spp. (Fig. 2). The antimicrobial activity of the strains was shown to be proportional to the level of gene expression (Fig. 10).

Microcin is a bacteriocin produced by Gram-negative bacteria, usually under stress conditions, with a low molecular mass (below 10 kDa) and high stability against pH changes, temperature changes, and protease activity (6). Microcin E492 was first reported to be produced by the fecal-origin K. pneumoniae strain RYC492 (14, 15). It is an 84-amino-acid siderophore peptide with a molecular mass of 8,717 Da that is produced, modified, and secreted by genes on the chromosome (6). Our study found that the microcin E492 gene was remarkably prevalent in ST23 isolates (Fig. 1 and 2). K. pneumoniae ST23 has been reported worldwide as an important hypervirulent clone that is usually associated with severe clinical outcomes such as pyogenic liver abscess, bacteremia, meningitis, or brain abscess (16). The high prevalence of microcin E492 among ST23 strains, in combination with its strong effect and wide spectrum of activity, may contribute to the pathogenicity and successful growth dynamics of this clone within the microbial flora. On the other hand, microcin E492 was also present in non-ST23 isolates (ST105 and ST161 [Fig. 1]), which was different from the findings in another study by Struve et al. (17). Struve et al. reported that the microcin E492-encoding gene was not identified in any non-clonal complex 23 (CC23)-related isolates and that the genomic island encoding three virulence factors (microcin E492, yersiniabactin, and colibactin) was exclusive to CC23-related strains. From our data, microcin E492 coexisted with yersiniabactin and colibactin only in ST23 isolates and not in ST105 and ST161 strains. Gene alignment displayed the presence of mobile elements and high homology of the upstream regions of *mceA* among MS6116 (ST161), MS5424 (ST105), and RYC492 (ST35) (Fig. 3), indicating that the microcin E492 gene clusters of these non-ST23 strains were inserted into the chromosomes at the same location. In contrast, the upstream regions of microcin E492 loci were diverse between ST23 isolates (Fig. 3), suggesting their insertion into different locations on the chromosome of the ST23 lineage.

Interestingly, in contrast to microcin E492, all the other microcin types and colicin-like bacteriocins were present in non-ST23 isolates, except microcin B17. Microcin B17 and microcin S are plasmid-encoded microcins that have previously been identified only in E. coli (13, 18); this is the first report of the existence of these microcins in K. pneumoniae, despite the low frequencies (0.6% and 1.7% within the studied population, respectively), suggesting the possible rare horizontal transfer of this bacteriocin-harboring plasmid between E. coli and KpSC. The two variants of microcin S from our two K. pneumoniae strains and one *K. quasipneumoniae* strain carried some mutations compared to that of E. coli G3/10, indicating that recombination events occurred during the transfer of the bacteriocin genes.

Colicin-like bacteriocins, particularly cloacin-like, displayed a weaker effect and narrower spectrum of activity than did microcin E492 (Fig. 2, 11, and 12), except in the case of K. aerogenes, where cloacin-like exhibited high inhibitory activity while microcin E492 showed almost no effect. The narrow spectrum of colicin activity has been explained by the specific receptors on the surface of the target strains (7). The action of colicin includes the following three steps: the binding of colicin to a specific receptor on the outer membrane (for example, BtuB, Tsx, OmpF, IutA, FepA, Cir, or FhuA), the translocation of colicin through the cell envelope to its target by either the Tol or TonB machinery, and the killing action. All colicins carry a three-domain structure, including the central, N-terminal, and C-terminal domains, which correspond to receptor binding, translocation, and killing activity, respectively (7). Cloacin is a colicin-like bacteriocin that has been shown to have RNase activity. In our study, amino acid sequence analysis showed 5 variants of cloacin, with mutations also frequently occurring at the C-terminal region rather than the other regions. The C terminus is associated with killing activity, and the variations in this area may explain the different activities. The expression level is also a factor contributing to the different inhibitory effects. In contrast to some microcins, such as class IIb, which have been found to be chromosome encoded, all reported colicins are plasmid derived, and the colicin gene clusters seem to be highly conserved (6). From plasmid comparison, we also found high conservation of the CloDF13 cluster and the cloacin-like gene clusters, including the cloacin gene, immunity protein gene, and colicin release lysis protein gene, among two of our strains (Fig. 7).

Klebicin B-like was the second most common type among the six bacteriocins detected; however, 82.4% of them produced unfunctional protein due to the disruption or truncation of the *kba* gene. Analysis showed that the gene expression of klebicin B-a (in MS6116 and MS6110) was higher than that of the disrupted klebicin B (in MS6163 and MS5265) (Fig. 10), which may be due to the loss of *kba* promoter in disrupted *kba* (Fig. 7). Nevertheless, the three isolates with putative intact *kba* genes did not display antibacterial activity, which may be caused by a defective function of the klebicin B protein owing to the low homology with *kba* from pKlebB-k17/80 or by the decreased expression due to mutations at the promoter regions (Fig. 7 and Fig. S1).

Regarding the klebicin C-like bacteriocin, the high similarity of the *kcp* gene suggested the similar binding capacity of these bacteriocins to the target bacterial host. The proteins encoded by *kcp* and *kdp* have been reported to be similar to the C-terminal domain of several tail fiber proteins, whose functions were comparable to those of the receptor binding domains of colicins (19). However, the low similarity of *kca* and *kci*, which encode the activity protein and immunity protein, respectively, may indicate the different recombination patterns during the evolution and may explain the different inhibitory effects.

When investigating the inhibitory activity against 21 AMR K. pneumoniae, certain indicator strains showed resistance or weak susceptibility against the microcin group (6/21 strains [28.6%]) and the colicin group (10/21 strains [47.6%]) (Fig. 9). Some indicator strains also carried cloacin-like or klebicin B-like, and therefore, they may possess the immunity factor against the respective bacteriocin groups. In other cases, mutations in the bacteriocin receptors, in the protein associated with bacteriocin translocation, or in the targets of the bacteriocin mechanism could lead to a resistance phenotype. Resistance to microcin B17 in E. coli involves mutations in the outer membrane porin OmpF, in the inner membrane transporter protein SbmA, or in the target of antimicrobials GyrB (20, 21). Mutations in *tonB*, *exbB*, and *semA* are associated with low susceptibility to MccE492 in E. coli K-12 (22). Over 70% of E. coli isolates were reported to have resistance against one or more colicins (23). Four mechanisms associated with colicin resistance have been detected, including (i) a plasmid-encoded immunity factor, (ii) alteration of the colicin receptor on the cell surface, (iii) alteration of the cell membrane proteins related to colicin translocation, and (iv) lysogen superinfection exclusion, which prevents repeat infections by the same phage (23).

The potential ecological role of microcins and colicins has suggested the application of bacteriocin-producing strains as probiotics. For instance, the application of the MccC7/C51-producing E. coli H22 strain led to the inhibition of Shigella flexneri in a mouse model (24). Some bacteriocin-producing strains with high antibacterial activity found in our study, especially those with a low-virulence profile and high activity against MDR bacteria, e.g., the non-ST23 microcin E492-carrying strains or the low-virulence cloacin-carrying strains, could become candidates as probiotic agents in future studies. In addition, the high susceptibility of some ESBL- or carbapenemase-producing K. pneumoniae to microcin E492 suggested the potential application of this microcin as a novel antimicrobial therapy for infections caused by MDR K. pneumoniae. Further studies should help clarify the toxicity of this microcin *in vivo* and define how it could contribute to infection prevention.

A limitation of this work is that the high proportion of HMV isolates (170/180) may not reflect the bacteriocin composition of the general KpSC population. However, our data showed that the bacteriocin prevalence is strongly linked to the ST composition but not to the HMV phenotype. Among the five most prevalent STs in this HMV population, including ST23, ST65, ST86, ST268, and ST37 (12), 88.9% of ST23 isolates carried microcin E492 and 77.8% of ST37 isolates carried cloacin or klebicin B, whereas the ST65, ST86, and ST268 lineages were 100% negative for all bacteriocin types (Fig. 1), indicating that not all HMV-associated STs carried bacteriocin and that bacteriocin positivity is not related to HMV phenotype but is associated with ST or clonality.

In summary, our study clarified the distribution of bacteriocin genes among 180 KpSC members based on genome sequence analysis. Some strains carrying multiple variants of bacteriocins were identified, and the different antibacterial patterns of each type have been demonstrated. Our findings suggest that the existence of different bacteriocin-carrying KpSC strains may affect the bacterial composition of the surrounding microbial community.

## MATERIALS AND METHODS

### Bacteriocin gene identification based on whole-genome sequences.

The Illumina whole-genome sequences of 180 clinical KpSC isolates (including 170 HMV and 10 non-HMV strains), which were isolated from patients from different hospitals in the Kansai and Chugoku regions of Japan (DDBJ BioProject numbers PRJDB12075, DRX309353 to DRX309536, and DRX317549 to DRX317564) (12), were used for genome analysis. The preliminary detection of gene clusters involved in the biosynthesis of ribosomally synthesized and posttranslationally modified peptides (RiPPs) and unmodified bacteriocins was conducted with the web server BAGEL4 (25). From the results of BAGEL4, bacteriocin genes of each isolate were extracted and confirmed with BLAST (https://blast.ncbi.nlm.nih.gov/Blast.cgi) to determine their precise nomenclatures. Strains showing no bacteriocin genes were excluded, and 59 isolates with at least one bacteriocin gene were included in the subsequent investigations.

### Subspecies identification, MLST, and MST analysis.

The subspecies identification and MLST of 180 KpSC isolates was investigated using Kleborate v2.1.0 (26). The allelic profile or ST was assigned based on the allelic number of seven loci in the order *gapA*, *infB*, *mdh*, *pgi*, *phoE*, *rpoB*, and *tonB*, in accordance with the KpSC MLST database (https://bigsdb.Pasteur.fr/klebsiella/). The connection between different STs was analyzed using PHYLOViZ v2.0 (27). As with the goeBURST full minimum spanning tree (MST), an MST was created to visualize the possible evolutionary relationships between STs, and information on bacteriocin types was overlaid to infer the distribution of bacteriocins among different ST populations.

### Phylogenetic tree construction by SNP analysis.

A phylogenetic tree was created based on whole-genome single nucleotide polymorphism (SNP) analysis using CSIPhylogeny 1.4 from the Center for Genomic Epidemiology with default settings (28). The phylogenetic tree was then annotated with Interactive Tree of Life (iTOL) (29).

### Amino acid sequence analysis of microcin S-like bacteriocin and cloacin.

Amino acid sequences similar to microcin S from two strains were identified and compared with the McsS sequence from E. coli G3/10 (13).

The amino acid sequences of cloacin-like from 13 isolates were extracted and compared with klebicin CCL and CloDF13 using BLAST (https://blast.ncbi.nlm.nih.gov/Blast.cgi). A phylogenetic tree based on the cloacin amino acid sequences was created using MEGA11 (30).

The amino acid sequences of klebicin B-like from 2 isolates were extracted and compared with klebicin B from pKlebB-k17/18 (GenBank accession number AF156893) using BLAST.

Amino acid sequence alignments were performed with MUSCLE algorithm using Snapgene (https://www.snapgene.com).

### Gene cluster comparison of microcin E492, microcin S, cloacin, klebicin B, and klebicin C-like bacteriocin.

The microcin E492 gene clusters from two ST23 isolates (MS6153 and MS6158) and two non-ST23 isolates (MS6116 and MS5424) were extracted and comparted with that from RYC492 (GenBank accession number APGM01000000) using EasyFig (31).

The microcin S-like gene clusters from three isolates (MS6116, MS6182, and MS6152) were extracted and compared with that from E. coli G3/10 (GenBank accession number JN887338) using EasyFig (31).

The MinION complete sequences of two strains, MS5288 and MS5538, that were obtained previously (12) were used for plasmid comparison. The cloacin-carrying plasmids of these two strains were identified and aligned with the publicly available plasmid CloDF13 (GenBank accession number X04466).

The klebicin B-like gene clusters from 17 isolates were extracted compared with that of plasmid pKlebB-k17/80 (GenBank accession number AF156893) using BLAST Ring Image Generator (BRIG) (https://brig.sourceforge.net) and EasyFig. The promoter regions of *bka* were aligned with the MUSCLE algorithm from Snapgene.

The gene clusters surrounding the klebicin C-like bacteriocin from two isolates (MS6111 and MS6250) were extracted and aligned with the publicly available klebicin C cluster (GenBank accession number AY578793) and klebicin D cluster (GenBank accession number AY578792).

### Bacterial strains and growth conditions.

The bacteriocin gene-positive KpSC isolates were cultured in Luria-Bertani (LB) broth (Becton, Dickinson and Company, Franklin Lakes, NJ, USA) and incubated at 37°C aerobically. Gram-negative indicator strains, including Klebsiella pneumoniae, Klebsiella quasipneumoniae, Klebsiella variicola, Klebsiella oxytoca, Klebsiella aerogenes, Escherichia coli, Pseudomonas aeruginosa, Pseudomonas fluorescens, Acinetobacter baumannii, Acinetobacter nosocomialis, Acinetobacter ursingii, Acinetobacter baylyi, Enterobacter cloacae, Stenotrophomonas maltophilia, Chryseobacterium indologenes, Serratia marcescens, and Morganella morganii, were cultured in LB broth at 37°C aerobically. Gram-positive indicator bacteria were cultured in Trypticase soy broth (TSB). Staphylococcus aureus and Staphylococcus epidermidis were grown at 37°C aerobically. Streptococcus mutans was grown at 37°C under 5% CO_2_. The origins of all strains are shown in Table S1 (32–36).

### Soft-agar overlay assay.

To evaluate the antibacterial activity of each bacteriocin, a soft-agar overlay assay was performed as previously described (37), with some modifications. Bacteriocin-producing strains were precultured in 3 mL of LB medium at 37°C aerobically for 6 h. All producer strains were equalized to an optical density at 660 nm (OD_660_) of 0.8 in 1.5-mL microcentrifuge tubes and inoculated onto LB plates (containing 1.5% agar) by means of a toothpick. The plates were incubated at 37°C for 16 h to create the 3-mm uniform diameter of the bacterial growth zone.

Indicator strains were precultured in 3 mL of LB medium at 37°C overnight. An aliquot of 100 μL of each indicator strain (OD_660_ = 1.0) was added to 3.5 mL of prewarmed half-strength LB soft agar (0.75%). Mixed overlay components were poured over the agar plates containing colonies of producers. The plates with S. mutans as an indicator were incubated at 37°C under 5% CO_2_ for 24 h, while the remaining plates were incubated at 37°C aerobically for 6 h. The diameters of the growth inhibition zones surrounding the bacteriocin producers were measured in three directions, and the mean value was calculated.

### Quantification of bacteriocin gene expression.

The cell pellets were harvested from colonies on LB agar plates. Briefly, an aliquot of 1 mL of overnight culture was inoculated onto LB agar and incubated at 37°C for 6 h before being collected by scraping the colonies. Total RNA (including microRNA [miRNA]) from the cell pellets was extracted using the miRNeasy minikit (Qiagen, Germany) according to the manufacturer’s recommended protocols. DNA contamination was removed from exactly 20 μg of extracted RNA samples using DNase I (Invitrogen, USA) before cleaning and concentrating the RNA samples with an RNeasy MinElute cleanup kit (Qiagen, Germany) according to the manufacturer’s specified protocol. The RNA purity and concentration were measured by a NanoDrop spectrophotometer.

One microgram of high-quality purified RNA was converted to cDNA in a 20-μL reaction volume using the Transcriptor first-strand cDNA synthesis kit (Roche, Switzerland) with the protocol recommended by the manufacturer. Quantitative PCR was performed with FastStart Essential DNA Green Master and LightCycler 96 instrument (Roche, Tokyo, Japan) using the cDNA as the template. The results were normalized to the gene *recA* and were analyzed with double delta threshold cycle (*C_T_*) analysis. The primers used in this study are listed in Table S2. Three independent experiments were performed and the average value was calculated.

### Data availability.

The data supporting the findings of this study are available within the article and its supplemental material.
