# Supplementary material for: Comprehensive Analysis of Bacteriocins Produced by the Hypermucoviscous Klebsiella pneumoniae Species Complex
Source: Microbiol Spectr. 2023 May 8;11(3):e00863-23. doi: 10.1128/spectrum.00863-23 (PMC10269471; doi:10.1128/spectrum.00863-23)
Supplement: Supplemental file 1 — Supplemental material. Download spectrum.00863-23-s0001.docx, DOCX file, 0.5 MB [file spectrum.00863-23-s0001.docx]

Supplemental Table S1. Indicator strains used in this study

|  | **Species** | **Strain name** | **Origin** | **Antimicrobial resistance** | **AMR genes** | **Source** |
| --- | --- | --- | --- | --- | --- | --- |
| 1 | *Klebsiella pneumoniae* | MS5288 | Clinical isolate | Hv-AMR | *bla*_IMP-6_, *bla*_CTX-M-2_, *aadA2*, *aacA4'*, *sul1*, *tetA* | [1] |
| 2 | *Klebsiella pneumoniae* | N2531 | Clinical isolate | Hv-AMR | *bla*_IMP-6_, *bla*_CTX-M-2_, *aadA2*, *aacA4'*, *sul1*, *tetA* | [1] |
| 3 | *Klebsiella pneumoniae* | N2576 | Clinical isolate | Hv-AMR | *bla*_IMP-6_, *bla*_CTX-M-2_, *aadA2*, *aacA4'*, *sul1*, *tetA* | [1] |
| 4 | *Klebsiella pneumoniae* | N454 | Clinical isolate | Hv-AMR | *bla*_IMP-6_, *bla*_CTX-M-2_, *aadA2*, *aacA4'*, *sul1*, *tetA* | [1] |
| 5 | *Klebsiella pneumoniae* | N579 | Clinical isolate | Hv-AMR | *bla*_IMP-6_, *bla*_CTX-M-2_, *aadA2*, *aacA4'*, *sul1*, *tetA* | [1] |
| 6 | *Klebsiella pneumoniae* | N212 | Clinical isolate | HMV-AMR | *bla*_IMP-6_, *bla*_CTX-M-2_, *aadA2*, *aacA4'*, *sul1*, *tetA*, *qnrS1*, *dfrA1* | [1] |
| 7 | *Klebsiella pneumoniae* | MS5292 | Clinical isolate | HMV-AMR | *bla*_IMP-6_, *bla*_CTX-M-2_, *aadA2*, *aacA4'*, *sul1*, *tetA* | [1] |
| 8 | *Klebsiella pneumoniae* | N60 | Clinical isolate | HMV-AMR | *bla*_IMP-6_, *bla*_CTX-M-2_, *aadA2*, *aacA4'*, *sul1*, *tetA*, *qnrS2* | [1] |
| 9 | *Klebsiella pneumoniae* | MS5293 | Clinical isolate | HMV-AMR | *bla*_IMP-6_, *bla*_CTX-M-2_, *aadA2*, *aacA4'*, *sul1*, *tetA, GyrA-83F, GyrA-87N, ParC-80R* | [1] |
| 10 | *Klebsiella pneumoniae* | MS5294 | Clinical isolate | HMV-AMR | *bla*_IMP-6_, *bla*_CTX-M-2_, *aadA2*, *aacA4'*, *sul1*, *tetA, GyrA-83F, GyrA-87N, ParC-80R* | [1] |
| 11 | *Klebsiella pneumoniae* | MS5265 | Clinical isolate | HMV-AMR | *bla*_IMP-6_, *bla*_CTX-M-2_, *aadA2*, *aacA4'*, *sul1*, *tetA, GyrA-83F, GyrA-87N, ParC-80R* | [1] |
| 12 | *Klebsiella pneumoniae* | MS5291 | Clinical isolate | HMV-AMR | *bla*_IMP-6_, *aadA2*, *aacA4'*, *sul1*, GyrA-83F, GyrA-87N, ParC-80R | [1] |
| 13 | *Klebsiella pneumoniae* | MS5538 | Clinical isolate | HMV-AMR | *bla*_KPC-2_, *bla*_CTX-M-65_, *aadA2*, *aph3-Ia*, *rmtB*, GyrA-83I, GyrA-87G, ParC-80I, *catA2*, *tetA*, *dfrA1*, *fosA* | [1] |
| 14 | *Klebsiella pneumoniae* | MS5544 | Clinical isolate | HMV-AMR | *bla*_KPC-2_, *bla*_CTX-M-65_, *aadA2*, *aph3-Ia*, *rmtB*, GyrA-83I, GyrA-87G, ParC-80I, *catA2*, *tetA*, *dfrA1*, *fosA* | [1] |
| 15 | *Klebsiella pneumoniae* | MS5784 | Clinical isolate | HMV-AMR | *bla*_CTX-M-15_*, strA*, *strB*, GyrA-83I, ParC-80I, *qnrB1*, *sul2*, *catA2*, *tetA, dfrA14* | [1] |
| 16 | *Klebsiella pneumoniae* | MS5783 | Clinical isolate | HMV-AMR | *bla*_CTX-M-15_*, strA*, *strB*, GyrA-83I, ParC-80I, *qnrB1*, *sul2*, *catA2*, *tetA, dfrA14* | [1] |
| 17 | *Klebsiella pneumoniae* | MS6104 | Clinical isolate | HMV-AMR | *bla*_CTX-M-15_*, aacA4'*, *qnrB1*, *catB4*, *tetA, dfrA14* | [1] |
| 18 | *Klebsiella pneumoniae* | MS6127 | Clinical isolate | HMV-AMR | *bla*_SHV-27_ | [1] |
| 19 | *Klebsiella pneumoniae* | MS6217 | Clinical isolate | HMV-AMR | *bla*_SHV-27_ | [1] |
| 20 | *Klebsiella pneumoniae* | MS6077 | Clinical isolate | HMV-AMR | *bla*_SHV-27_ | [1] |
| 21 | *Klebsiella pneumoniae* | MS6080 | Clinical isolate | HMV-AMR | *bla*_SHV-27_ | [1] |
| 22 | *Klebsiella pneumoniae* | MS5286 | Clinical isolate |  |  | This study |
| 23 | *Klebsiella quasipneumoniae* | MS5285 | Clinical isolate | AMR |  | This study |
| 24 | *Klebsiella quasipneumoniae* | MS6123 | Clinical isolate |  |  | This study |
| 25 | *Klebsiella variicola* | MS6176 | Clinical isolate | AMR |  | This study |
| 26 | *Klebsiella variicola* | MS6154 | Clinical isolate |  |  | This study |
| 27 | *Klebsiella oxytoca* | MS5390 | Clinical isolate | AMR |  | This study |
| 28 | *Klebsiella aerogenes* | 12C-A | Clinical isolate | AMR |  | [2] |
| 29 | *Escherichia coli* | K12 | Clinical isolate, Standard strain |  |  | ATCC |
| 30 | *Escherichia coli* | 77E | Clinical isolate | AMR | *bla*_CTX-M-27_, *aph(3")-Ib*, *aph(6)-Id*, *mdf(A)*, *mph(A), sul1*, *sul2*, *tetA* | [2] |
| 31 | *Pseudomonas aeruginosa* | PAO1 | Clinical isolate, Standard strain |  |  | ATCC |
| 32 | *Pseudomonas aeruginosa* | 71E | Clinical isolate | AMR | *bla*_IMP-1_, *bla*_TEM-1B_, *aadA1*, *aac(6)-Iae*, *aph(3')-II*, *sul1*, *catB7*, *fosA* | [2] |
| 33 | *Pseudomonas fluorescens* | 59C-A | Clinical isolate |  |  | [2] |
| 34 | *Pseudomonas fluorescens* | 82C | Clinical isolate |  |  | [2] |
| 35 | *Acinetobacter baumanii* | 25E | Clinical isolate |  |  | [2] |
| 36 | *Acinetobacter nosocomialis* | 70C | Clinical isolate | AMR |  | [2] |
| 37 | *Acinetobacter ursingii* | 56C | Clinical isolate | AMR | *bla*_CARB-2_, *aac(6')-Ib3, aph(3")-Ib*, *aph(3')-Ia*, *aph(6)-Id*, *aac(6')-Ib-cr*, *mrs(E)*, *mph*(E), *floR*, ARR-3, *sul1*, *sul2*, *tet(39)*, *dfrA17* | [2] |
| 38 | *Acinetobacter baylyi* | 68E | Clinical isolate |  |  | [2] |
| 39 | *Enterobacter cloacae* | 82E | Clinical isolate | AMR |  | [2] |
| 40 | *Stenotrophomonas maltophilia* | 20C | Clinical isolate |  |  | [2] |
| 41 | *Chryseobacterium indologenes* | 60E | Clinical isolate |  |  | [2] |
| 42 | *Chryseobacterium indologenes* | 65E-B | Clinical isolate |  |  | [2] |
| 43 | *Serratia marcescens* | 102C-B | Clinical isolate |  |  | [2] |
| 44 | *Morganella morganii* | 18C-A | Clinical isolate | AMR |  | [2] |
| 45 | *Staphylococcus aureus* | MW2 | Clinical isolate | MRSA |  | CDC |
| 46 | *Staphylococcus aureus* | COL | Clinical isolate | MRSA |  | [3] |
| 47 | *Staphylococcus aureus* | RN4220 | Clinical isolate |  |  | [4] |
| 48 | *Staphylococcus epidermidis* | KSE1 | Clinical isolate |  |  | [5] |
| 49 | *Staphylococcus epidermidis* | KSE5 | Clinical isolate |  |  | [5] |
| 50 | *Streptococcus mutans* | UA159 | Clinical isolate, Standard strain |  |  | [6] |
| Hv; hypervirulent, HMV; hypermucoviscous, AMR; antimicrobial resistant, MRSA; methicillin resistant *Staphylococcus aureus*, ATCC; American Type Culture Collection, CDC; Centers for Disease Control and Prevention | | | | | | |

**References**

1. ﻿Le MN-T, Kayama S, Wyres KL, Yu L, Hisatsune J, Suzuki M, Yahara K, Terachi T, Sawa K, Takahashi S, Okuhara T, Kohama K, Holt KE, Mizutani T, Ohge H, Sugai M. 2022. Genomic epidemiology and temperature dependency of hypermucoviscous *Klebsiella pneumoniae* in Japan. Microb Genom 8:mgen000827. https://doi.org/10.1099/mgen.0.000827

2. ﻿Le MN-T, Kayama S, Yoshikawa M, Hara T, Kashiyama S, Hisatsune J, Tsuruda K, Onodera M, Ohge H, Tsuga K, Sugai M. 2020. Oral colonisation by antimicrobial-resistant Gram-negative bacteria among long-term care facility residents: prevalence, risk factors, and molecular epidemiology. Antimicrob Resist Infect Control 9:45. https://doi.org/10.1186/s13756-020-0705-1.

3. ﻿Kornblum J, Hartman BJ, Novick RP, Tomasz A. 1986. Conversion of a homogeneously methicillin-resistant strain of *Staphylococcus aureus* to heterogeneous resistance by Tn551-mediated insertional inactivation. Eur J Clin Microbiol 5:714–718. https://doi.org/10.1007/BF02013311.

4. ﻿Kreiswirth BN, Löfdahl S, Betley MJ, O’Reilly M, Schlievert PM, Bergdoll MS, Novick RP. 1983. The toxic shock syndrome exotoxin structural gene is not detectably transmitted by a prophage. Nature 305:709–712. https://doi.org/10.1038/305709a0.

5. ﻿Nakazono K, Le MN-T, Kawada-Matsuo M, Kimheang N, Hisatsune J, Oogai Y, Nakata M, Nakamura N, Sugai M, Komatsuzawa H. 2022. Complete sequences of epidermin and nukacin encoding plasmids from oral-derived *Staphylococcus epidermidis* and their antibacterial activity. PLoS One 17:e0258283. https://doi.org/10.1371/journal.pone.0258283

6. ﻿Murchison HH, Barrett JF, Cardineau GA, Curtiss R. 1986. Transformation of *Streptococcus mutans* with chromosomal and shuttle plasmid (pYA629) DNAs. Infect Immun 54:273–282. https://doi.org/10.1128/iai.54.2.273-282.1986.

Supplemental Table S2. Primers used in this study

| **Gene name** | **Product name** | **Forward and Reverse Primers** | | **PCR objective** |
| --- | --- | --- | --- | --- |
| *mceA* | Microcin E492 | F R | 5’-gattaggatcagcagctttg-3’ 5’-aactggatgttgcgctgt-3’ | RT-PCR |
| *mcsS* | Microcin S | F R | 5’-atgtcaaaagtaagagagttaa-3’ 5’-ggtgcattacgtcctaa-3’ | RT-PCR |
| *mcbA* | Microcin B17 | F R | 5’-gcgagtgaatttggtgtag-3’ 5’-tccaccactacaaccgtt-3’ | RT-PCR |
| *ccl* | Cloacin | F R | 5’-ctcatgaatatggctccg-3’ 5’-ctggataaggattcattcatt-3’ | RT-PCR |
| *kba* | Klebicin B | F R | 5’-gacctcgattttgacgata-3’ 5’-tccagttattgccaacgt-3’ | RT-PCR |
| *kca* | Klebicin C-a | F R | 5’-gtacttagcgacccgttag-3’ 5’-ttcgacaacgtttcactg-3’ | RT-PCR |
| *kca* | Klebicin C-b | F R | 5’-ggcatgactagcgttaatct-3’ 5’-agttgcttcatcacgaact-3’ | RT-PCR |
| *recA* | Recombinase A | F R | 5’-gatcgacgaaaacaaacaga-3’ 5’-atatccagagaaagcgagc-3’ | RT-PCR, internal control |

Supplemental Figure S1. Comparison of klebicin B amino acid sequences
